# A Rapid Method for the Detection of Sarcosine Using SPIONs/Au/CS/SOX/NPs for Prostate Cancer Sensing

**DOI:** 10.3390/ijms19123722

**Published:** 2018-11-22

**Authors:** Dagmar Uhlirova, Martina Stankova, Michaela Docekalova, Bozena Hosnedlova, Marta Kepinska, Branislav Ruttkay-Nedecky, Josef Ruzicka, Carlos Fernandez, Halina Milnerowicz, Rene Kizek

**Affiliations:** 1 Department of Research and Development, Prevention Medicals, Tovarni 342, 742 13 Studenka-Butovice, Czech Republic; dagmar.uhlirova@seznam.cz (D.U.); MartStan@seznam.cz (M.S.); MichaelaDocekalova@seznam.cz (M.D.); ruzicka.josef@iol.cz (J.R.); 2 Department of Human Pharmacology and Toxicology, Faculty of Pharmacy, University of Veterinary and Pharmaceutical Sciences Brno, Palackeho 1946/1, 612 42 Brno, Czech Republic; bozena.hosnedlova@post.cz (B.H.); brano.ruttkay@seznam.cz (B.R.-N.); 3 Department of Biomedical and Environmental Analyses, Faculty of Pharmacy with Division of Laboratory Diagnostics, Wroclaw Medical University, Borowska 211, 50-556 Wroclaw, Poland; zalewska.m@gmail.com (M.K.); halina.milnerowicz@umed.wroc.pl (H.M.); 4 School of Pharmacy and Life Sciences, Robert Gordon University, Garthdee Road, Aberdeen AB10 7QB, UK; c.fernandez@rgu.ac.uk

**Keywords:** superparamagnetic iron oxide nanoparticles, gold nanoparticles, biosensor, biomarker, Trinder reaction, personalized medicine

## Abstract

Background: Sarcosine is an amino acid that is formed by methylation of glycine and is present in trace amounts in the body. Increased sarcosine concentrations in blood plasma and urine are manifested in sarcosinemia and in some other diseases such as prostate cancer. For this purpose, sarcosine detection using the nanomedicine approach was proposed. In this study, we have prepared superparamagnetic iron oxide nanoparticles (SPIONs) with different modified surface area. Nanoparticles (NPs) were modified by chitosan (CS), and sarcosine oxidase (SOX). SPIONs without any modification were taken as controls. Methods and Results: The obtained NPs were characterized by physicochemical methods. The size of the NPs determined by the dynamic light scattering method was as follows: SPIONs/Au/NPs (100–300 nm), SPIONs/Au/CS/NPs (300–700 nm), and SPIONs/Au/CS/SOX/NPs (600–1500 nm). The amount of CS deposited on the NP surface was found to be 48 mg/mL for SPIONs/Au/CS/NPs and 39 mg/mL for SPIONs/Au/CS/SOX/NPs, and repeatability varied around 10%. Pseudo-peroxidase activity of NPs was verified using sarcosine, horseradish peroxidase (HRP) and 3,3′,5,5′-tetramethylbenzidine (TMB) as a substrate. For TMB, all NPs tested evinced substantial pseudo-peroxidase activity at 650 nm. The concentration of SPIONs/Au/CS/SOX/NPs in the reaction mixture was optimized to 0–40 mg/mL. Trinder reaction for sarcosine detection was set up at 510 nm at an optimal reaction temperature of 37 °C and pH 8.0. The course of the reaction was linear for 150 min. The smallest amount of NPs that was able to detect sarcosine was 0.2 mg/well (200 µL of total volume) with the linear dependence *y* = 0.0011*x* − 0.0001 and the correlation coefficient *r* = 0.9992, relative standard deviation (RSD) 6.35%, limit of detection (LOD) 5 µM. The suggested method was further validated for artificial urine analysis (*r* = 0.99, RSD 21.35%, LOD 18 µM). The calculation between the detected and applied concentrations showed a high correlation coefficient (*r* = 0.99). NPs were tested for toxicity and no significant growth inhibition was observed in any model system (*S. cerevisiae*, *S. aureus*, *E. coli*). The hemolytic activity of the prepared NPs was similar to that of the phosphate buffered saline (PBS) control. The reaction system was further tested on real urine specimens. Conclusion: The proposed detection system allows the analysis of sarcosine at micromolar concentrations and to monitor changes in its levels as a potential prostate cancer marker. The whole system is suitable for low-cost miniaturization and point-of-care testing technology and diagnostic systems. This system is simple, inexpensive, and convenient for screening tests and telemedicine applications.

## 1. Introduction

### 1.1. Basal Metabolism

**Figure 1:**
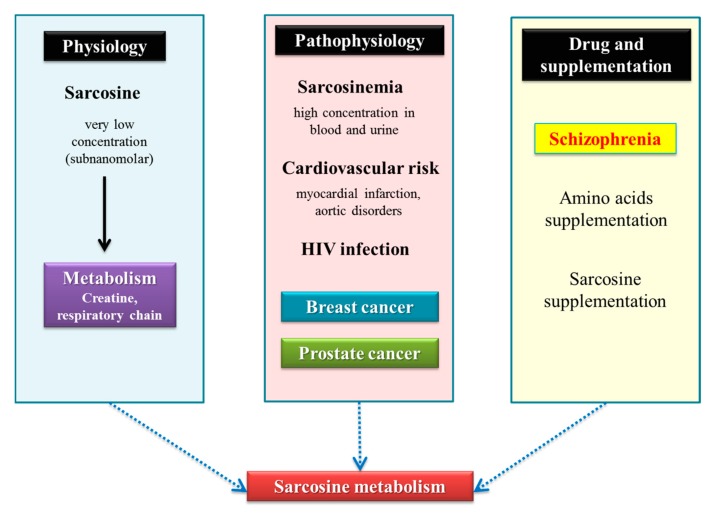
The physiological, pathophysiological role of sarcosine including its use as a food supplement or a drug. Sarcosine is detected in healthy people in very low concentrations and primarily is involved in the metabolism of creatine [4] and respiratory chain [5]. Several pathophysiological roles of sarcosine have been described, especially its elevated levels due to sarcosine dehydrogenase deficiency (sarcosinemia) [1] and also there are studies suggesting its importance in infarction, or aortic disorders [6]. A study suggested that plasma sarcosine could also be used as metabolic biomarker of HIV/AIDS infection [7]. Changes in sarcosine levels in urine are significantly associated with prostate tumors and may also represent specific targeting to tumor tissue. The addition of sarcosine was also used for the treatment of schizophrenia [8]. Sarcosine (*N*-methylglycine) is an endogenous antagonist of glycine transporter-1, which potentiates glycine’s action on *N*-methyl-d-aspartate glycine site and can have beneficial effects on schizophrenia [9].

Sarcosine (*N*-methylglycine; CH_3_NHCH_2_COOH), a key intermediate in one-carbon metabolism [1], is enzymatically formed from dimethylglycine by dimethylglycine dehydrogenase (DMGHD; EC 1.5.99.2) [2] and is generated from glycine by glycine-*N*-methyl transferase (GNMT; EC 2.1.1.20) [3]. Sarcosine is formed from dietary intake of choline and from the methionine metabolism, and is rapidly degraded [4] by the sarcosine dehydrogenase (SARDH; EC 1.5.99.1) [1] to glycine, which plays a substantial role in many physiological processes as a main metabolic source of components of living cells, such as glutathione, creatine, purines and serine [4]. SARDH is linked by electron-transferring flavoprotein (ETF) to the respiratory redox chain [5]. Thus, sarcosine is a normal physiological metabolite and its concentration in blood serum of humans is reported to be 1.4 ± 0.6 μM [4]. Increased levels of sarcosine have been found in various diseases (Figure 1).

### 1.2. Sarcosinemia and Sarcosinuria

Mutations in the gene encoding sarcosine dehydrogenase (SARDH) are associated with an autosomal recessive disorder—sarcosinemia (OMIM 268900)—which is manifested by elevated levels of sarcosine in blood and urine. In the SARDH, four different mutations (P287L, V71F, R723X, R514X) were identified. In addition, uniparental disomy in the region of SARDH was found [1]. Sarcosinemia and sarcosinuria also occur in some patients with type II glutaric aciduria and with severe deficiency of folic acid [2].

### 1.3. Homocysteine Metabolism

The metabolic pathway of sarcosine is linked to the metabolic pathway of betaine [10]. Large oral doses of betaine have proved effective in lowering plasma homocysteine in severe hyperhomocysteinaemia [11]. It was suggested that administration of precursors of methyl groups via *N*^5^-methyltetrahydrofolate (*N*^5^-MTHF), such as betaine or products of one-carbon metabolism (dimethylglycine, sarcosine, l-serine, or glycine), could be beneficial for patients suffering from homocystinuria. Betaine as a specific methyl donor is involved in the conversion of l-homocysteine to l-methionine [12]. After betaine administration, homocysteine level decreased and methionine level increased by providing an additional pathway for homocysteine transmethylation to methionine. During betaine addition, all patients experienced an increase in the ratio of plasma methionine/total homocysteine, regardless of estimated methionine intakes, compliance with methionine restriction or betaine [13].

### 1.4. Prostate Tumors

Prostate cancer (PCa) is the second most common cancer and the fifth leading cause of death from cancer in men [14]. Despite the fact that the five-year survival rate for PCa patients with localized tumors exceeds 95%, patients with metastases have a total five-year survival less than 30% [15]. The gold standard for PCa diagnosis is the analysis of a prostate specific antigen (PSA) level in serum, but this biological marker is influenced by other factors besides progression or development of PCa [16]. PSA levels provide a valuable metric for PCa detection, however, the reliability of PSA as a surrogate biomarker for progression of this disease has recently been questioned [17]. Therefore, searching for other biomarkers associated with tumors has a key importance [15,18,19,20,21,22,23,24] and several of these have progressed into the clinical laboratory. In the glycine-*N*-methyl transferase *(GNMT*) gene, a 1289 C→T polymorphism was associated with cancer risk [25]. *GNMT* was down-regulated in most of the prostatic tumor specimens. Immunohistochemical staining displayed an abundant *GNMT* expression in normal prostatic and benign prostatic hyperplasia tissues compared with the PCa tissue [26]. It is known that epigenetic changes can be linked with carcinogenesis [27]. Altered methylation in some genes has been reported to be involved in the pathogenesis of PCa [28,29]. *GNMT* expression is decreased in PCa tissue and the loss of heterozygosity in the *GNMT* promoter region is associated with PCa [26]. Huang et al. [26] analyzed 4-bp insertion/deletion polymorphisms (INS/DEL) of *GNMT* and revealed that haplotype A (16GAs/DEL/C) carriers had significantly higher risk for PCa in comparison to haplotype C (10GAs/INS/T) individuals [26]. In addition, it was revealed that T allele of the rs9462856 single-nucleotide polymorphism (SNP) in the promoter region of the *GNMT* gene is overexpressed in patients suffering from PCa [30]. *GNMT* regulates *S*-adenosylmethionine levels [31] and thus is a metabolic link between de novo synthesis of methyl groups and the availability of dietary methionine [26]. The *GNMT* enzyme influences DNA methylation by affecting de novo synthesis of methyl groups. [31] *GNMT*, by affecting DNA methylation, influences genetic stability [26]. Furthermore, research studies substantiated the role of sarcosine in PCa progression [3,32]. Sreekumar et al. [3] identified sarcosine as a potential mediator of PCa progression. Sarcosine levels were also increased in invasive PCa cell lines relative to benign prostate epithelial cells. This differential metabolite can be non-invasively detected in urine [3]. Metabolomic profiling of PCa progression identified substantially increased levels of sarcosine in metastatic PCa and moderate but significant elevation of the metabolite in PCa urine [32].

### 1.5. Nanoscale Materials in Cancer Diagnosis

The use of various nanostructures for different biological applications has been widely described [33,34,35,36,37,38,39,40,41]. A number of novel, highly sensitive diagnostic platforms [42] have recently been developed to measure biomolecules [42,43,44,45,46], with a great promise for early disease detection [42]. Systems based on gold nanoparticles (AuNPs) have been previously used for sensing amino acids [47,48,49]. Biosensing strategies based on magnetic nanoparticles (NPs), due to their unique properties such as financial affordability, high physical and chemical stability [42], displaying negligible background signal in biological specimens [50], and biocompatibility [39,42], have attracted great attention. Moreover, biological samples evince essentially no magnetic background, therefore, magnetic NPs can be used for biosensing applications, and highly sensitive measurements can be performed in visually obscured samples [42]. So far, numerous methods have been developed to sense biomolecules using magnetic labels [51,52]. In our previous study, superparamagnetic iron oxide nanoparticles (SPIONs) were used for an ultrasensitive specific biosensing system to detect sarcosine [53]. The great advantages of magnetic NPs in bioanalysis are their easy surface functionalization [53], a combination of the molecular-recognition-specific binding of analytes to their surface [54] and the specific elimination of undesirable (magnetically responsive) biomolecules adsorption from complex sample mixtures [55,56]. In this study, we used AuNPs combined with SPIONs, whose surface was modified with several different molecules. The aim of this work was to propose a highly sensitive and selective method for urinary sarcosine detection using enzyme modified SPIONs.

The SPION-based detection method for sarcosine appears to be an appropriate alternative for the colorimetric method which is clinically routinely used for the determination of a variety of biomolecules. Both methods are based on the Trinder reaction [57,58,59]. In addition, in contrast to the colorimetric method [60] the nanotechnology approach has the advantage of easy manipulation and the use of inflow technologies such as microchips.

## 2. Results

### 2.1. Preparation and Physicochemical Characterization of Nanoconstructs

**Figure 2:**
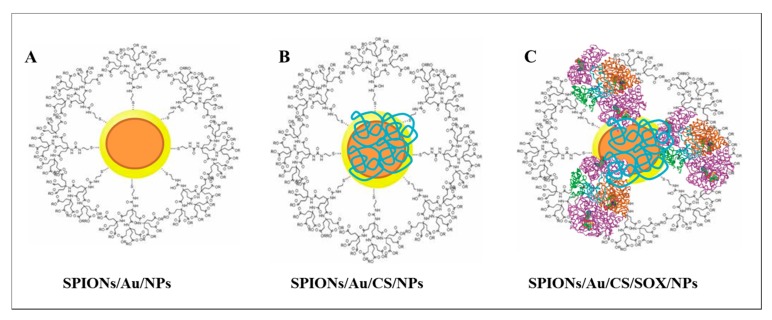
Simplified scheme of prepared superparamagnetic iron oxide nanoparticles (SPIONs). (**A**) SPIONs/Au/NPs with the surface modified with citric acid; (**B**) SPIONs/Au/CS/NPs modified with chitosan (CS); (**C**) SPIONs modified with CS and sarcosine oxidase (SOX)—SPIONs/Au/CS/SOX/NPs. The nanoparticles (NPs) thus prepared were characterized in detail by physicochemical methods. See main text and Section 4 for experimental details.

We have prepared three types of differently modified SPIONs combined with AuNPs. The NP surface was modified either with citric acid, chitosan (CS), or CS and sarcosine oxidase (SOX) (see in Figure 2). The amount of SOX captured on the surface of the SPIONs showed 30–40% of the original enzymatic activity.

**Figure 3:**
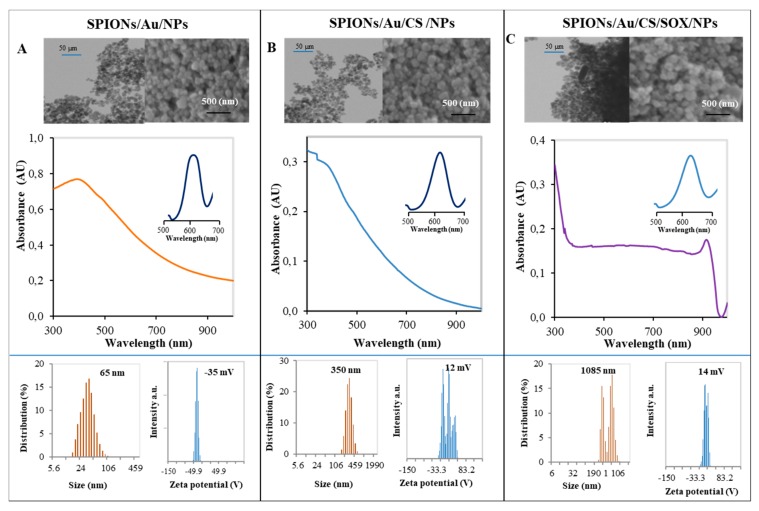
Physicochemical characteristics of SPIONs used. Scanning electron microscopy (SEM) analysis, typical absorbance spectra, pseudo-peroxidase activity, size distribution and zeta potential of NPs: (**A**) SPIONs/Au/NPs; (**B**) SPIONs/Au/CS/NPs; (**C**) SPIONs/Au/CS/SOX/NPs (1 kU/L SOX). Top of the figure—SEM photographs of prepared nanoparticles (NPs)—an overview photo (on the left) and close-up of a photo (on the right) of SPIONs. Middle of the figure—typical absorbance spectra and pseudo-peroxidase activity. Spectrophotometric measurements were carried out in 50 mM phosphate buffer, pH 8.0 in a UV cuvette filled with 100 μL of NPs and 1000 μL of 50 mM phosphate buffer. The small graphs above the graphs of absorbance vs. wavelength represent pseudo-peroxidase activity of the respective SPION particles after 5 min reaction (3,3′,5,5′-tetramethylbenzidine (TMB) reduction). Bottom of the figure—distribution diagrams of the size and graphs of the dependence of the signal intensity on zeta potential of SPIONs. The amount of SPIONs used was 40 mg/mL. Other experimental details are in Section 4.

The obtained particles were characterized by physicochemical methods. Physicochemical characteristics of the prepared SPIONs are shown in Figure 3. The morphology of the used NPs was determined by scanning electron microscopy (SEM). SEM images showed a homogeneous spherical NP population with a diameter of about 25 nm. The NP size obtained by the SEM analysis was described as follows: SPIONs/Au/NPs: 18–22 nm, SPIONs/Au/CS/NPs: 21–25 nm, and SPIONs/Au/CS/SOX/NPs: 24–28 nm. The size (aggregate size) distribution profile of the NPs was also measured by dynamic light scattering (DLS) method with the following values: SPION/Au/NPs—10–100 nm (with a mean of 65 nm), SPIONs/Au/CS/NPs—250–450 nm (with a mean of 350 nm), and SPIONs/Au/CS/SOX/NPs—300–1200 nm (with a mean of 1085 nm). SPIONs/Au/NPs evinced a negative charge (−10 to −40 mV) (with a mean of −35 mV), whereas in SPIONs/Au/CS/NPs and SPIONs/Au/CS/SOX/NPs, the charge was positive—0 to 20 mV (with a mean of 12 mV) and 10 to 40 mV (with a mean of 14 mV), respectively. The maxima of absorbance spectra observed for all the studied particles were found at about 530 nm. As shown in Figure 3, absorbance spectra of all three types of SPIONs have the usual course. SOX enzyme loaded onto the SPIONs is stable and transportable.

### 2.2. Pseudo-Peroxidase Activity of the Gold SPIONs

**Figure 4:**
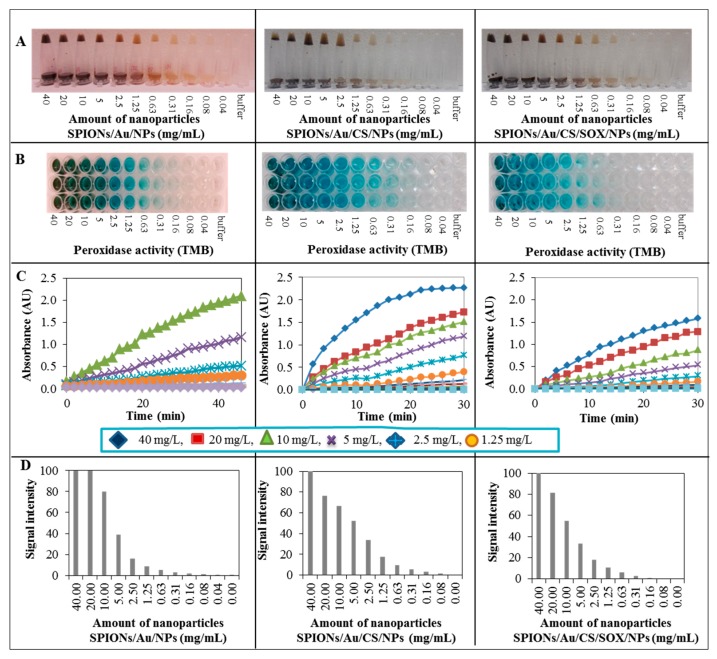
Pseudo-peroxidase activity of SPIONs/Au/NPs, SPIONs/Au/CS/NPs, SPIONs/Au/CS/SOX/NPs. Photographs (camera Canon PowerShot SX610 HS, 20.2 Mpx CMOS sensor, ultra-wide-angle lens with 18× superzoom) showing (**A**) a typical appearance of individual used types of SPIONs in a range of concentrations from 0 to 40 mg/mL in 50 mM phosphate buffer (pH 8.0) and (**B**) a typical color course of pseudo-peroxidase reaction with SPIONs/Au/NPs in a range of concentrations from 0 to 40 mg/mL; representative image; (**C**) time course of pseudo-peroxidase reaction (0 to 30 min) for SPIONs/Au/NPs in a range of concentrations from 0 to 10 mg/mL; SPIONs/Au/CS/NPs and SPIONs/Au/CS/SOX/NPs (0–40 mg/mL); (**D**) Signal intensity of SPIONs/Au/NPs, SPIONs/Au/CS/NPs and SPIONs/Au/CS/SOX/NPs in dependence on pseudo-peroxidase reaction signal in a range of concentrations from 0 to 40 mg/mL. Composition of reaction mixture for the measurement of pseudo-peroxidase activity was as follows: 5 mM TMB, hydrogen peroxide 30%, 0.5 M acetate buffer pH 4. The measurement was carried out in the Brand plate after washing with 18 MΩ water, at 650 nm in 1 min reading interval, each value being the average of five repeated measurements. Other experimental details are in Section 4.

Pseudo-peroxidase activity of all three prepared types of SPIONs is demonstrated in Figure 4. Figure 4A shows images of characteristic appearance of individual types of SPIONs used depending on their amount. Further, a typical color changed from colorless to shades of blue (Figure 4B). The time course with change of absorbance (Figure 4C) of pseudo-peroxidase reaction with SPIONs/Au/NPs can be seen. In addition, the signal intensity of SPIONs/Au/NPs in dependence on the amount of NPs (Figure 4D), can also be observed.

### 2.3. Characterization of SPIONs Using Ninhydrin Assay

**Figure 5:**
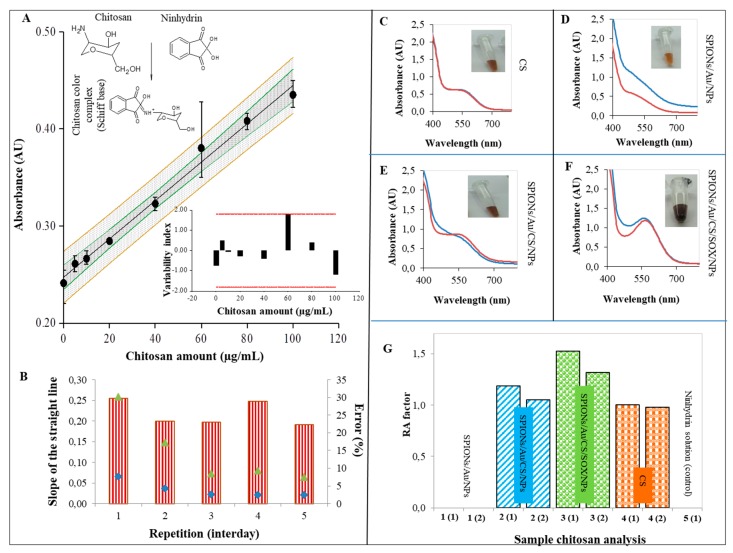
Ninhydrin assay for evaluation of different modifications of SPIONs. (**A**) Calibration curve of chitosan (CS) in a ninhydrin assay. The calibration curve was made from five independent repeats with 95% confidence band. In the inset, the variability of the individual values with the confidence band as a variability index; (**B**) the independent repetition of the calibration curve shown in (**A**) with a slope. The left vertical axis of the graph measures the slope of the straight lines of the repetitions from five independent days (red-white striped columns). The right vertical axis measures the RSD error of calculation of the backward concentration (green triangles) and the average error from the standard deviation of individual measurements of the repeat point variability (blue diamonds). It was calculated by fitting the values into the calibration curve. A typical absorption spectrum of ninhydrin complex in two independent preparations (blue and red line), with a photographic illustration (20.2 Mpx): (**C**) CS (100 μg/mL); (**D**) SPIONs/Au/NPs; (**E**) SPIONs/Au/CS/NPs; (**F**) SPIONs/Au/CS/SOX/NPs) (**G**) RA ninhydrin factor of the prepared SPIONs in two independent preparations. Measurements were performed in a 96-well microplate as a scan. Blank was a ninhydrin reagent. Sample was prepared as following: 10 mL of nanoparticles (NPs) was incubated with ninhydrin (20 mg/mL) reagent for 30 min at 100 °C. RA ninhydrin factor was calculated as the ratio of 100 µg/mL CS signal/CS signal of NPs. Error bars were calculated from 5 independent measurements.

For detection of CS adsorbed to NP surface, the ninhydrin assay was used. The scheme in Figure 5A demonstrates the principle of ninhydrin reaction with CS to characterize CS NPs. The calibration curve of CS in a ninhydrin assay (Figure 5A) was also constructed in independent repetitions for five days (Figure 5B). The color intensity of the ninhydrin complex formed was spectrophotometrically evaluated as a measure of depolymerized CS activity. A typical absorption spectrum of ninhydrin complex in the case of CS alone is shown in Figure 5C. The absorption spectra of individual types of prepared SPIONs are depicted in Figure 5D–F). Relative absorbance (RA ninhydrin factor) of each prepared CS-coated SPIONs solution was calculated as follows: RA = absorbance of 100 µg/mL CS solution/absorbance of NPs solution (Figure 5G). The amount of CS on SPIONs/Au/CS/NPs and SPIONs/Au/CS/SOX/NPs surfaces was found to be 48 mg/mL and 39 mg/mL respectively, and repeatability varied around 10%.

### 2.4. Characterization of SPIONs for Sarcosine Detection—SOX Activity of SPIONs/Au/CS/SOX/NPs

**Figure 6:**
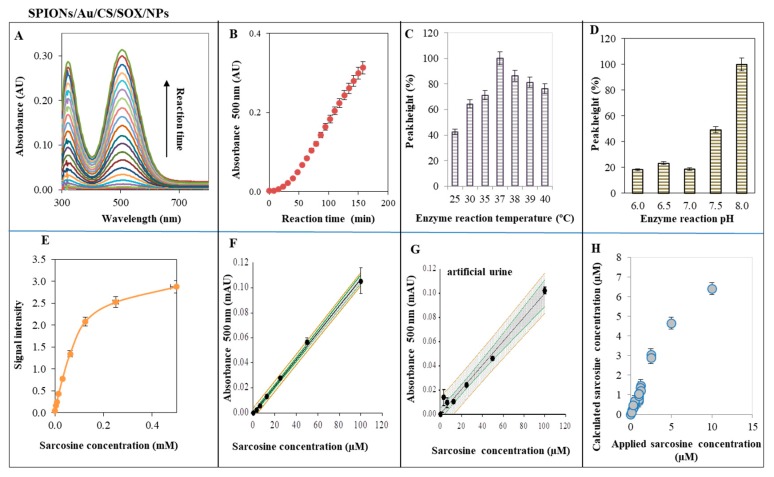
Determination of SOX activity of SPIONs/Au/CS/SOX/NPs using 4-AAP. 4-AAP reaction dependence of time reaction course. (**A**) Typical absorbance spectra (350–800 nm), time measurements, the first time is zero and measurements were repeated after 3 min up to 60 min and can be seen how the curve rises (i.e., last green line is measurement after 60 min; the penultimate red is measurement after 57 min, and so forth); (**B**) 4-AAP reaction dependence of the signal at 500 nm on reaction time of the main signal; (**C**) 4-AAP reaction dependence on the temperature (25, 30, 35, 37, 38, 39, 40 °C) of the enzyme reaction; (**D**) 4-AAP reaction dependence on pH (6.0, 6.5, 7.0, 7.5, 8.0) effect of the enzyme reaction. Dependence of signal intensity: (**E**) on high sarcosine concentration (0–0.5 mM); and (**F**) low concentrations of sarcosine (0–100 μM); (**G**) sarcosine in artificial urine (0–100 μM); (**H**) correlation between the applied and calculated concentration of sarcosine (0–10 μM). Error bars were calculated from 5 independent measurements. Other details are provided in Section 4.

SOX activity of SPIONs/Au/CS/SOX/NPs was performed using 4-aminoantipyrine (4-AAP) (Figure 6). The absorbance spectra were characteristic as can be seen in Figure 6A. Figure 6B illustrates the dependence of absorbance on the reaction time course. We tested the effect of the reaction temperature and pH (Figure 6C,D) for determining the optimal conditions for SOX activity and then the final conditions for the preparation of urine specimens and for the detection of sarcosine. Figure 6E,F represent the dependence of signal and absorbance, respectively, on various concentrations of sarcosine. Even at lower concentrations of sarcosine (to 100 μM), the reaction was sensitive leading to the emergence of the signal (Figure 6E), whereas at higher concentrations of sarcosine (to 0.5 mM), the absorbance increased linearly (Figure 6F). The absorbance linear curve showed a good reproducibility. The course of the calibration curve in artificial urine is shown in Figure 6G. Figure 6H illustrates the comparison between applied and calculated concentration of sarcosine from the calibration curve.

**Figure 7:**
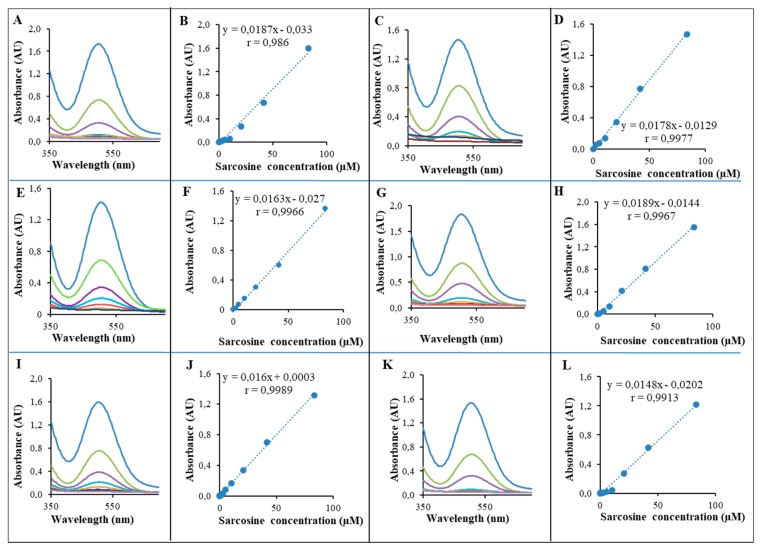
SOX activity of SPIONs/Au/CS/SOX/NPs depending on the concentration of the NPs. Various amounts of SPIONs/Au/CS/SOX/NPs (0.2, 0.4; 0.8; 1.2; 1.6; 2.0 mg/well) were pipetted into the microtiter plate. (**A**) Absorbance scan on various concentrations of sarcosine; (**B**) dependence signal TMB on various concentrations of sarcosine; 2.0 mg of nanoparticles (NPs) per well; (**C**) absorbance scan on various concentrations of sarcosine; (**D**) dependence signal TMB on various concentration of sarcosine; 1.6 mg of NPs per well; (**E**) absorbance scan on various concentrations of sarcosine; (**F**) dependence signal TMB on various concentrations of sarcosine; 1.2 mg of NPs per well; (**G**) absorbance scan on various concentrations of sarcosine; (**H**) dependence signal TMB on various concentrations of sarcosine; 0.8 mg of NPs per well; (**I**) absorbance scan on various concentrations of sarcosine; (**J**) dependence signal TMB on various concentrations of sarcosine; 0.4 mg of NPs per well; (**K**) absorbance scan on various concentrations of sarcosine; (**L**) dependence signal TMB on various concentrations of sarcosine; 0.2 mg of NPs per well. Spectrophotometric measurements of SOX activity were performed at temperature of 23 °C for 30 min after the addition of sarcosine. They were aspirated to dryness, then 125 μL of colored substrates (4-AAP, phenol, triton X-100, pH 8 phosphate) and 25 μL of various sarcosine concentrations in water (0, 0.4, 0.8, 1.6, 3.1, 25, 50, 100 μg/mL) were added. All measurements were performed in five replicates in the range of 350–700 nm for scans and 510 nm for concentration dependencies. For graphs A, C, E, G, I and K each line represents one concentration of nanoparticles (blue is 2.0 mg/well, green is 1.6 mg/well, purple is 1.2 mg/well, light blue is 0.8 mg/well, etc.) For other details see Section 4.

SOX assay with various amounts of SPIONs/Au/CS/SOX/NPs (0.2–2.0 mg/well) is presented in Figure 7A–L. For each of the six tested concentrations, a good calibration curve was obtained. The obtained dependencies were linear (*r* = 0.98–0.99), with RSD in the range of 5–15% depending on the amount of NPs added. The detection system was also very reproducible in the presence of a very small amount of modified NPs.

### 2.5. Assessing Toxicity of SPIONs

**Figure 8:**
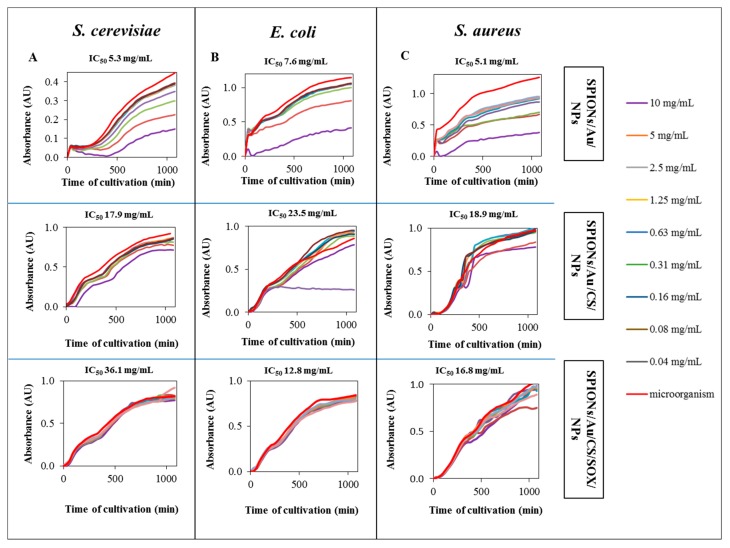
Toxicity study of individual investigated types of SPIONs performed in (**A**) eukaryotic cells *S. cerevisiae*; and prokaryotic cells (**B**) *E. coli*; (**C**) *S. aureus*. 100 µL of the microorganism culture and 50 µL of nanoparticles (NPs) were pipetted into the 96-microwell plate and then 50 µL of pure medium added. The cultures were grown for 16 h at 37 °C. Time scans of optical density (600 nm) were measured at regular intervals (15 min) and average five times measurement. Other experimental details are in Section 4.

Toxicity of SPIONs was tested on both eukaryotic model organism *S. cerevisiae*, and microorganisms—Gram negative (*E. coli*) and Gram positive (*S. aureus*) species of bacteria (Figure 8). For toxicity determination, the half maximal inhibitory concentration (IC_50_) was calculated. IC_50_ for SPIONs/Au/NPs ranged for each biological model within the range of values from 5.1 to 7.6 mg/mL (Figure 8A–C). However, SPIONs modified with both CS and SOX were compared to non-modified SPIONs far less toxic to the yeast and bacterial models used. As shown in Figure 8A, a modification with CS and SOX markedly decreased (almost seven times) toxicity in *S. cerevisiae*, compared to control SPIONs (without any modification), whereas in both bacteria species, the highest value of IC_50_ was found in CS-stabilized SPIONs. Toxicity of SPIONs/Au/CS/NPs compared to SPIONs/Au/NPs in *E. coli* and *S. aureus* was found to be 3.1 and 3.7 times lower, respectively (Figure 8B,C). Thus, CS and SOX modification prolonged inhibition of biological processes.

### 2.6. Analysis of SPIONs Hemolytic Properties In Vitro

**Figure 9:**
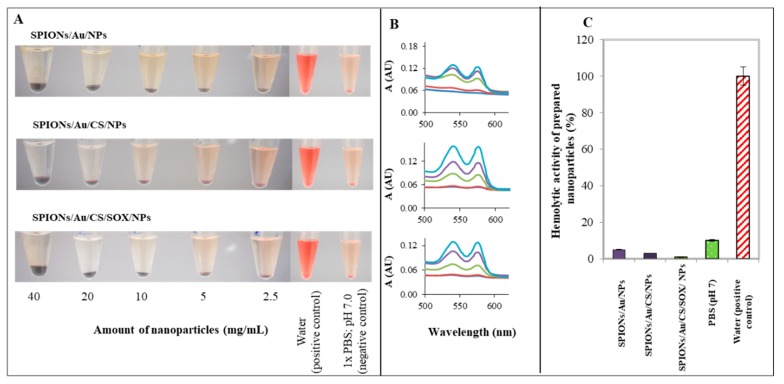
Determination of hemolytic activity of prepared nanoparticles (NPs). (**A**) Photographs of each variant (centrifugation 2000× *g*, 5 min to a completely clear solution). Tested variants: water (positive control), 1× PBS, pH 7.0 (negative control), SPION/Au/NPs, SPION/Au/CS/NPs, SPION/Au/CS/SOX/NPs (40, 20, 10, 5, 2.5 mg/mL). (**B**) Supernatant was spectrophotometrically analyzed, the absorbance spectra were obtained and the results were evaluated at 570 nm (light blue line is a positive control, dark blue line is a negative control, and violet, green and red lines represent 40, 20, 10, 5, and 2.5 mg/mL of NPs, respectively. (**C**) Relative comparison of hemolytic activity of tested NPs, 100% of water is a positive control. Experiment was carried out in four replications. Blood parameters were: RBC (red blood cells) 5.16 × 10^12^ L; HBG (hemoglobin) 161 g/L; MCV (mean corpuscular volume) 83.6 fL. Error bars were calculated from 5 independent measurements. For other details see Section 4.

Hemolytic activity of each type of SPION was tested at various concentrations in the range 2.5–40 mg/mL (Figure 9A), with water and PBS as a positive and negative control, respectively. The in vitro hemolysis assay was performed on human erythrocytes and calculated based on the above-mentioned formula (see Section 4). The SPION respective of absorbance spectra curves (Figure 9B) and graph of hemolytic activity (Figure 9C) showed a very low toxicity to red blood cells.

### 2.7. Application of SPIONs for Urine Sample Analysis

**Figure 10:**
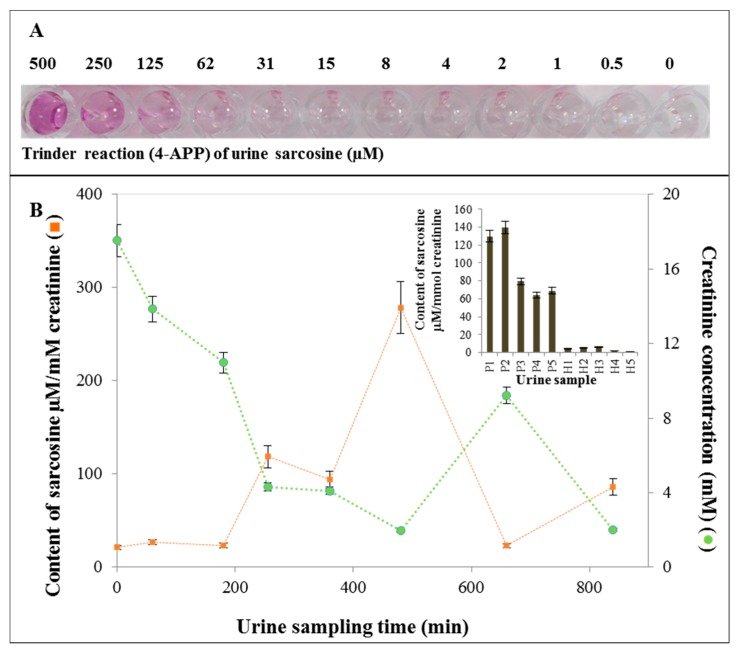
Application of nanoparticles (NPs) for urine sample analysis. (**A**) A typical course of SOX color reaction at various applied sarcosine concentrations in urine specimen; (**B**) Time changes in urine sarcosine levels after peroral application (1.5 g). The small bar graph: Sarcosine levels in urine specimens from donors with PCa (P1, P2, P3, P4, P5) and donors without PCa (H1, H2, H3, H4, H5). Error bars were calculated from 5 independent measurements. The amount of sarcosine was converted to creatinine (more details are given in Section 4).

Based on SOX assay and artificial urine analysis, we applied the obtained findings to real urine samples, and subsequently, sampling with sarcosine secreted over time (Figure 10B). Trinder reaction with 4-AAP at various concentrations applied into urine sarcosine resulted in the formation of sarcosine concentration-dependent colored product as depicted in Figure 10A. Figure 10B illustrates the content of sarcosine converted to mmol of creatinine level in patient samples as well as healthy controls. Changes in urine sarcosine levels after administration in time showed the highest value after 480 min. Figure 10B shows that the sarcosine content ranged from 1 to 6 µM/creatinine mM (mean 3.6 µM/creatinine mM) in healthy volunteers, whereas, in PCa patients ranged around 64–140 µM/creatinine mM (with a mean of 96.4 µM/creatinine mM). Thus, the content of sarcosine in the urine of PCa patients is about 27 times higher than in healthy people. Furthermore, our previous study [61] revealed that the level of urinary sarcosine in healthy patients is very low, and its level in patients suffering from PCa is several times higher than that of cured patients.

## 3. Discussion

### 3.1. Preparation and Physicochemical Characterization of Nanoconstructs

NPs are unique tools for very efficient and effective separation and for monitoring biological processes. Nanomedical usability of such NPs dramatically increases due to their potential in enhancing extremely sensitive and selective diagnostics of serious diseases including cancer. The SPIONs (γ-Fe_2_O_3_ and Fe_3_O_4_) are multi-purpose nanoscaled materials that have been used in various applications [62,63,64]. SPIONs consist of a solid core made up of iron oxides (magnetite, Fe_3_O_4_ and/or maghemite, Fe_2_O_3_) mostly coated with biocompatible polymers [65]. They can be easily functionalized to provide binding sites for various types of molecules [53]. The coating agent plays a double role: it protects NPs from oxidation and enhances their stability when dispersed in aqueous media [65]. SPIONs were used in our previous study, where we introduced an ultrasensitive specific biosensing system for detection of sarcosine as a potential biomarker of PCa [53]. In this study, we used them in combination with AuNPs and modified them with citric acid, and we used CS and SOX as surface coatings (Figure 2). CS, due to its excellent film-forming ability, high permeability, ease of chemical modification, mechanical strength, non-toxicity, and low cost, is considered to be one of the most-used biopolymers for immobilization of biomolecules. In addition, −NH_2_ groups of CS provide the hydrophilic environment for the biomolecules [66]. SOX has been recently used for improvement of amperometric biosensor for detection of creatinine [67] and sarcosine [68]. Narwal et al. [68] described the construction of an amperometric sarcosine biosensor based on covalent immobilization of SOX onto the nanocomposite of carboxylated multi-walled carbon nanotubes/CS and copper NPs (CS/CuNPs/MWCNT) modified gold electrode with better analytic performance. AuNPs have been also recently used for development of sensing system for sarcosine detection [69,70]. The size of prepared SPIONs (Figure 3) obtained by SEM analysis (18–28 nm) showed relatively narrow size distributions which are in accordance with the studies of Hirsch et al. [71] that reported the size of 28.3 ± 2.1 nm in polyvinyl alcohol (PVA)-coated SPIONs (PVA-SPIONs), and of Park et al. [72] that measured the size ranging from 22.4 to 25.1 nm in polymer-coated SPIONs. Mardinoglu and Cregg et al. [73] used three different sizes of SPIONs coated with oleic acid with diameters 6.6, 11.6, and 17.8 nm, and Kapri et al. [74] synthesized SPIONs with size in the range of 10.6–37.8 nm (10.6, 20.0 and 37.8 nm). Herve et al. [75] reported the size of PEG[poly(ethylene glycol]ylated SPIONs to be 82.4 ± 7.9 nm. Moreover, the size of particles grew with the degree of surface functionalization—with each coating the size increased (18–22 vs. 21–25, and 24–28 nm for SPIONs/Au/NPs, SPIONs/Au/CS/NPs, and SPIONs/Au/CS/SOX/NPs, respectively). This result is consistent with the observation of Herve et al. [75] that found the average hydrodynamic size of PEGylated NPs to be higher than those of the initial and silanized SPIONs. The increase in hydrodynamic size of NPs was consistent with PEG coating [75]. For comparison, the NPs PEGylated using covalent surface binding by Xie et al. [76] exhibited a size distribution with a narrow maximum at 70 nm as measured by DLS. Zeta potential measurement was also performed to determine the potential stability and polarity of the NPs [65]. The physical stability is one of the most critical requirements for a NP system [77]. The zeta potential value is a characteristic that points to the storage stability of colloidal dispersions [78]. A high zeta potential (+ or −) value reflects the electrostatic interaction within a NP system and is a feature of the dispersion stability of SPIONs [79]. In the present study, the zeta (ζ) potential of SPION/Au/NPs, SPIONs/Au/CS/NPs, and SPIONs/Au/CS/SOX/NPs was found to be −35, 12 and 14 mV, respectively (Figure 3A–C). After the SPIONs/Au/NPs synthesis, the negative charge typical for AuNPs [80,81,82] remained (zeta potential from −10 to −40 mV). In the remaining two types of NPs, the charge was positive: for SPIONs/Au/CS/NPs zeta potential ranged from 0 to 20 mV, and for SPIONs/Au/CS/SOX/NPs from 10 to 40 mV, which may be attributed to positive charge on surface of NPs due to the presence of functional groups of surface coating, such as –NH_2_ group of CS. De Palma et al. [83] measured negative values of zeta potential at pH values over the range 3–9 in magnetic NPs with amino endgroups, in contrast to those with carboxyl groups. The amino groups exhibit an isoelectric point (pI) of 10.4, which provides a continuous positive charge over the entire lower pH range due to the presence of NH_3_^+^ groups. The differences in zeta potential are affected by many factors, especially by a type of surface coating and pH of the solution [84]. However, the characterization of the different charged SPIONs of one type (neutral, positive, negative PVA-SPIONs) clearly showed that only the zeta potential varies between each NP type, whereas the particle sizes remain comparable [71]. As shown in Figure 3, in the presence of SOX, the character of the absorbance spectrum changed.

### 3.2. Pseudo-Peroxidase Activity of the Gold SPIONs

Due to their large surface-to-volume ratio, nanomaterials are attractive to be used as high-efficiency catalysts [85], and some of them have been found to have enzyme-like activity. To date, a variety of NPs, especially those formed from noble metals, have been determined to possess oxidase-, peroxidase-, catalase-, and/or superoxide dismutase-like activity [86]. Various research manuscripts on nanomaterials deal with their peroxidase. [87,88,89,90,91,92,93,94,95] or oxidase-like activity [96]. Pseudo-peroxidase activity of the AuNPs is very intensively investigated [97,98]. AuNPs are known to possess intrinsic biological peroxidase-like activity [90,99] that makes them appropriate for biosensor development [99]. In addition, Xu et al. [100] found that gold NPs bound on microgel particles could enhance the performance of horseradish peroxidase. In addition, AuNPs are observed to enhance the activities of glucose oxidase [101]. In conclusion, considering the high peroxidase-like activity, good cytocompatibility and ease of preparation, CS-AuNPs have a wide range of potential applications in biocatalysis and bioassays [97]. Gao et al. [88] found that Fe_3_O_4_ magnetic NPs possessed intrinsic enzyme mimetic activity similar to that found in natural peroxidases, though Fe_3_O_4_ magnetic NPs are thought to be biologically and chemically inert and they were subsequently used as peroxidase mimetics for H_2_O_2_ and glucose detection [93]. The peroxidase-like activity of Fe_3_O_4_ magnetic NPs has been also recently used for the development of immunochromatographic strip (Nanozyme-strip) for the rapid and sensitive detection of the Ebola virus glycoprotein [102]. Fang et al. [103] used Fe_3_O_4_/reduced graphene oxide (rGO) nanocomposites as the peroxidase mimic to prepare the modified glassy carbon electrode for electrochemical sensing of H_2_O_2_. In our study, we investigated the catalytic properties of the fabricated gold SPIONs (Figure 4). Functionalized AuNPs are the most prevalent gold surface biomarker detection platform [104]. The initial experimental characterization of AuNPs has been carried out in our previous study [105]. Furthermore, we have published physicochemical characterization of gold magnetic NPs (AuNPs) [106]. We are very intensively engaged in the pseudo-peroxidase activity of AuNPs. In this work, the peroxidase-like activity was evaluated in the catalytic oxidation of peroxidase substrate 3,3′,5,5′-tetramethylbenzidine (TMB), with the addition of H_2_O_2_ and acetate buffer. The proposed procedure was used to maximize the amplification of the resulting colored product (TMB). TMB substrate show a very good course of reaction depending on the type of AuNPs used. The TMB cation free radical is a one-electron oxidation product, which is formed by exposing TMB to peroxidase and H_2_O_2_. In addition, the TMB cation free radical is responsible for the blue color formed during the TMB oxidation [107]. The catalytic reaction was detected by monitoring absorbance change of oxidation products of TMB at 650 nm, similarly as reported in the study of Lv and Weng [108]. This work observed the peroxidase activity of hemin-graphene-gold (H-RGO-Au) ternary composite, leading to TMB oxidation at 652 nm. The same maximum absorbance was reported also by Marquez et al. [107] The catalytic activity of SPIONs was illustrated by catalyzing the oxidation reaction of TMB accompanied with a color change. As can be seen in Figure 4B, the color change is dependent on the concentration of TMB. The ability of SPIONs to oxidize TMB substrate accompanied by color reaction is shown in Figure 4B.

### 3.3. Characterization of SPIONs Using Ninhydrin Assay

We used ninhydrin assay to monitor CS properties in the presence of sodium tripolyphosphate (TPP), which has been used in our previous study [109], in which we have investigated the effects of some factors (the reaction temperature, reaction time and the ninhydrin concentration) on the CS-ninhydrin reaction to optimize the specificity and sensitivity. TPP stabilizes CS crosslinked structure and provides NP formation [109]. Ninhydrin assay was used for quantitative determination of CS by ninhydrin [110]. CSs are linear binary heteropolysaccharides composed of (1–>4)-linked 2-acetamido-2-deoxy-β-d-glucopyranose (GlcNAc) and 2-amino-2-deoxy-β-d-glucopyranose (GlcN) [110]. The relaxed structure of CS has an accessible amino group for ninhydrin reaction (Figure 5A). When TPP is added, the structure of CS is strongly crosslinked leading to the NP formation. The number of accessible amino groups decreases proportionally with the level of crosslinking and the structure of NPs formed. This behavior results in less accessible amino groups which causes lower absorbance signal of ninhydrin [110,111]. The color intensity of the ninhydrin complex was spectrophotometrically evaluated as a degree of depolymerized CS activity. The absorption spectra of ninhydrin complex for CS along with all three types of prepared SPIONs were characteristic (Figure 5C–F). According to a previous study, only the GlcN units of CSs are responsible for creating colored products with ninhydrin [110]. Firstly, the absorbance of ninhydrin was investigated in the presence of TPP. From Figure 5A,B is obvious that the equal signal intensity for all applied concentrations is comparable to ninhydrin signal intensity at A_572_. Further results are interleaved by the signal intensity of the CS alone (A_572_). Figure 5B shows the good repeatability of the directives of the lines of Figure 5A in individual days. Figure 5C demonstrates a decrease in the signal of the formed NPs in CS (Figure 5D–F). The very good reproducibility (Figure 5G) can be further assessed by looking at the bar graph of the RA factor (the result is always in two repeats) calculated according to the Equation 1 illustrated in Section 4.

### 3.4. Characterization of SPIONs for Sarcosine Detection—SOX Activity of SPIONs/Au/CS/SOX/NPs

Sarcosine was found to be significantly higher in urine sediments and supernatants derived from biopsy positive PCa patients as compared to biopsy negative controls [3]. Sarcosine was also reported to be a differential metabolite that is greatly increased during PCa progression [112]. In 2015, more than one million new prostate cancer patients were diagnosed [113]. Some studies have also reported measurement of sarcosine level in blood serum for diagnosis and medical management of PCa [68]. Sarcosine oxidase (SOX, EC 1.5.3.1) is a monomeric or heterotetrameric flavoprotein that catalyzes the oxidative demethylation of sarcosine (*N*-methylglycine) to yield glycine, formaldehyde, and hydrogen peroxide [114]. Monomeric SOX is often used with creatininase, creatinase, and horseradish peroxidase in enzymatic assays of creatinine and creatine in clinical settings [115]. The catalytic activity of the ferromagnetic NPs is dependent on pH, temperature and H_2_O_2_ concentration [116]. Directed evolution was used to expand the substrate specificity and functionality of SOX by using screening assay based on the 4-aminoantipyrine (4-AAP) peroxidase system (Figure 6). When used in the presence of phenol, 4-aminoantipyrine can be utilized to measure the peroxidase activity. Hydrogen peroxide is coupled with 4-aminoantipyrine and phenol in the presence of peroxidase to yield a chromogen (quinoneimine dye) with a maximum absorbance of at about 500 nm. This allows 4-aminoantipyrine to be used as a reagent in the determination of chemicals, in particular those in which the method of detection is coupled through peroxidase and other coupling enzymes. SOX assay using 4-APP reaction showed characteristic and reproducible absorbance spectra (Figure 6A). Figure 6B shows the slow linear course of the enzymatic reaction over time. We measured the peroxidase-like activity (510 nm) SPIONs/Au/CS/SOX/NPs while varying the pH from 6 to 8 at intervals of 0.5 and the temperature ranging from 25 to 40 °C as illustrated in Figure 6C,D, respectively. As evidenced by bar graphs (Figure 6C,D), the optimal pH and temperature were pH 8.0 and 37 °C, which are in good agreement to SOX activity of the enzyme solution used by Nishiya et al. [115] Thus, we adopted pH 8.0 and 37 °C as standard conditions for subsequent analysis of SPIONs/Au/CS/SOX/NPs activity. Even at lower concentrations of sarcosine (to 100 μM), the reaction was sensitive leading to the emergence of the signal (Figure 6E), whereas at higher concentrations of sarcosine (to 0.5 mM), the absorbance increased linearly with a good reproducibility (Figure 6F). The course of the calibration curve in artificial urine (Figure 6G) demonstrated that the calibration curve in urine was linear with very good reproducibility. The above procedure can be used for real urine sample analysis. Moreover, a high correlation value (*r*^2^ = 0.99) between the measured sarcosine value and the determined sarcosine concentration from the regression equation was obtained. It also shows the excellent reproducibility and applicability of the method for the real sample analysis (Figure 6H). Testing SOX activity with varying amounts of SPIONs/Au/CS/SOX/NPs (Figure 7A–L) has clearly shown that the proposed assay is also applicable for analyzing small amounts of particles, such as 0.2 mg/well (200 µL of total volume) (Figure 7L). This amount of NPs was able to detect sarcosine with the linear dependence *y* = 0.0011*x* − 0.0001 and the correlation coefficient *r* = 0.9992, RSD 6.35%, LOD 5 µM (Table 1). The suggested method was also validated for an artificial urine analysis (*r* = 0.9912, RSD 21.35%, LOD = 18 µM). The analytical parameters of both validations (in water and in artificial urine) are summarized in the Table 1.

### 3.5. Assessing Toxicity of SPIONs

The value of IC_50_ for SPIONs/Au/NPs in a *S. cerevisiae* eukaryotic model organism was found to be 5.3 mg/mL (Figure 8A). For comparison, AuNP-induced cytotoxicity in human tumor cells expressed as IC_50_ was higher than 15 μg/mL [117], i.e., more than 350 times lower than in our study. Smith et al. [118] found that yeast cell growth, assessed as cell yield, was unaffected by exposing intact cells to as much as 22 µM of the 0.8 nm AuNPs functionalized with positively charged *N*,*N*,*N*-trimethylammoniumethanethiol (TMAT), corresponding to the 100 µg/mL dose. Moreover, modification with CS and SOX led to an increase in IC_50_ value for yeast cells to 17.9 and 36.1 mg/mL, respectively (Figure 8B,C). CS is considered to be non-toxic for eukaryotic cells [119,120]. Pokharkar et al. [119] reported that CS-AuNPs did not cause any signs of intoxication after dosing to rats: the LD_50_ value was found to be greater than 2 mg/g. In addition, CSs (having different *M*w and DD; <5 kDa, 65.4% DD; 5–10 kDa, 55.3% DD; and >10 kDa, 55.3% DD) were found to display little cytotoxicity against CCRF-CEM (human lymphoblastic leukemia) and L132 (human embryonic lung cells) (IC_50_ >1 mg/mL) [121]. Moreover, CS was reported to possess hepatoprotective effects [122]. The increase in IC_50_ after SOX modification can be related to the fact that SOX is a protein involved in cell metabolism, and creates a protein corona that prevents from toxic effects. The IC_50_ values in non-modified AuNPs for prokaryotic model organisms *E. coli* and *S. aureus* were found to be 7.6 and 5.1 mg/mL, respectively, and similarly as for yeast, SPIONs modified with both CS and SOX were far less toxic (Figure 8B,C). Interestingly, in contrast to *S. cerevisiae*, CS caused a greater increase in IC_50_ value than SOX, because CS is widely known for its toxic effect to microorganisms either alone or blended with other natural polymers [123], and it exhibits toxicity to Gram-negative [124,125,126] as well as Gram-positive bacteria [124,125,127]. In our study, however, SPIONs/Au/NPs exhibited the most pronounced antibacterial activity against both test microorganisms, compared to SPIONs/Au/CS. IC_50_ in *E. coli* and *S. aureus* was found to be 3.1 and 3.7 times lower, respectively, for CS-modified SPIONs than for those without functionalization (Figure 8B,C). Thus, CS modification of gold SPIONs surprisingly prolonged inhibition of biological processes.

### 3.6. Analysis of SPIONs Hemolytic Properties In Vitro

Hemolysis is defined as the damage caused to red blood cells (RBCs), which results in the release of the iron-containing protein hemoglobin into plasma [128]. The small size and unique physicochemical properties of NPs may cause their interactions with erythrocytes to differ from those observed for conventional pharmaceuticals and may also cause interference with standardized in vitro tests. Separating true hemolytic responses from the false-positive or false-negative results caused by particle interference is important for correct interpretation of these tests [129]. Hemolysis assay was also used to assess the overall toxicity of prepared NPs. The hemocompatibility of the SPIONs was assessed by in vitro hemolysis assay (Figure 9A). The impact of NPs on human red blood cells lysis was carried out spectrophotometrically recording the absorbance of hemoglobin at 570 nm (Figure 9B). Each of SPIONs prepared evinced a very low hemolytic activity as can be seen in Figure 9B,C. The level of hemolytic activity declined with the degree of modification and it was found to be 5, 3 and 1% compared to positive control (deionized water) for SPION/Au/NPs, SPION/Au/CS/NPs, and SPION/Au/CS/SOX/NPs, respectively (Figure 9C). Thus, the highest hemolytic activity showed pristine (non-modified) gold SPIONs, whereas due to the formation of the protein corona by the SOX enzyme SPION/Au/CS/SOX/NPs exhibited five times lower toxicity to human erythrocytes. This finding corresponds very well to the IC_50_ testing of the NPs toxicity in eukaryotic model (Figure 8A). Our results are in accordance with results of another previous study, in which has been suggested that NP coating can reduce hemolytic toxicity of NPs [130]. This result is also in agreement with results of our previous study, in which the hemolytic activity of unmodified CS was observed to be 67%, and after CS NPs formation, hemolytic activity decreased by 30% [109]. Moreover, our findings confirm a high hemocompatibility of CS reported by Richardson et al. [121] that found that hemolysis was not observed (<10%) over 1 h and 5 h with CSs of <5 kDa, 5–10 kDa and >10 kDa at concentrations of up to 5 mg/mL.

### 3.7. Application of SPIONs for Sarcosine Analysis in Urine Samples

A number of methods have been developed for the detection of sarcosine [61,112,131,132]. Sarcosine levels in plasma of PCa patients were measured by fluorometric assay [112]. The content of sarcosine in PCa tissue samples was analyzed by gas chromatography/mass spectrometry (GC/MS) [131]. To determine sarcosine in urinary samples taken from patients suffering from PCa, high performance liquid chromatography (HPLC) with tandem MS, which is one of the most widely used analytical methods, was also used [133]. However, HPLC/MS may show inconsistent results among various authors, which may be due to the fact that alanine and sarcosine co-elute on an HPLC reversed-phase column and the mass spectrometer cannot differentiate between the two isomers [134]. Therefore, Meyer et al. [134] developed a reproducible and high-throughput HPLC/MS method to separate sarcosine from α- and β-alanine and to quantify sarcosine in human serum and urine. Wu et al. [135] applied isotope dilution gas chromatography/mass spectrometry (ID GC/MS) metabolomic approach combined with microwave-assisted derivatization (MAD) to analyze the urinary metabolomic information in PCa patients and participants with benign prostate hypertrophy and healthy persons. Biavardi et al. [136] introduced a cavitand-functionalized silicon surface as supramolecular receptor that was able to recognize sarcosine from its nonmethylated precursor, glycine, in water and urine. Bianchi et al. [137] developed a high-throughput method for the direct determination of sarcosine in urine and urinary sediments using hexyl chloroformate derivatization followed by direct immersion solid-phase micro extraction and fast gas chromatography–mass spectrometric analysis. Lan et al. [138] reported colorimetric determination of sarcosine in urine samples of prostatic carcinoma by mimic enzyme palladium NPs. We previously described an ultrasensitive specific biosensing system for detecting sarcosine based on Förster resonance energy transfer (FRET), employed for sarcosine quantification in prostatic cell lines (PC3, 22Rv1, PNT1A), and urinary samples of prostate adenocarcinoma patients [53]. As showed in our previous studies [61,132], the electrochemical detection can be considered as a convenient technique to detect sarcosine in very low concentration. However, this method can be used to analyze real samples only after application of appropriate pre-treatment [61,132]. In contrast, the method based on NPs developed in this study has been successfully tested on real urine specimens. In addition, it is very simple, rapid, sensitive, low-cost and repeatable.

## 4. Materials and Methods

### 4.1. Chemicals

Low molecular weight chitosan (CS), sodium tripolyphosphate (TPP) penta basic, dimethyl sulfoxide (DMSO), 3,3′,5,5′-tetramethylbenzidine (TMB), sodium citrate dihydrate, hydrogen tetrachloroaurate (III) tetrahydrate (HAuCl_4_·4H_2_O), hydrogen peroxide (H_2_O_2_), hydroxylammonium chloride (NH_2_OH·HCl), sodium carbonate anhydrous (Na_2_CO_3_), sodium hydrogen carbonate (NaHCO_3_), disodium hydrogen phosphate dodecahydrate (Na_2_HPO_4_·12H_2_O), sodium dihydrogenphosphate dihydrate (NaH_2_PO_4_·2H_2_O), acetic acid (HAc), and sodium acetate (NaAc) were all purchased from Merck. All reagents were of analytical grade and used without any further purification. Ninhydrin, hydrindatin were purchased from Ingos (Prague, Czech Republic). Aqueous solutions for size analysis were prepared using PURELAB^®^ Ultra (Elga, High Wycombe, United Kingdom) resistivity 18 mΩ-cm. For other purposes deionized water was used.

### 4.2. Synthesis of SPIONs/Au/NPs

1.3 g of Fe(NO_3_)_3_·9H_2_O was dissolved in 80 mL of water (18 MΩ). A quantity of 1.4 mL 25% NH_3_ was diluted in 8.6 mL water and 0.2 g NaBH_4_ was dissolved in this mixture and, subsequently, the mixture was stirred (300 rpm, 10 min at 22 °C). The solution color turned to dark brown. The mixture was heated to 100 °C for 2 h and stirred (300 rpm, stirring overnight at room temperature). The magnetic particles were separated from the solution via magnet and washed several times in water. The solution of HAuCl_4_ (20 mL, 1 mM) was added to the magnetic particles and stirred (300 rpm 3 h at 22 °C). Subsequently, the solution of C_6_H_5_Na_3_O_7_·2H_2_O (0.5 mL, 0.265 g/10 mL) was added to the mixture and stirred overnight. Finally, the gold SPIONs were separated via magnet and dried (40 °C).

### 4.3. Modification of SPIONs/Au/NPs with Chitosan (CS)

SPIONs/Au/NPs (40 mg) were washed 3 times with 1000 µL of 1× phosphate buffered saline (PBS) (pH 7.0), mixed using a vortex mixer for 60 s, and then separated using a neodymium magnet. Subsequently, SPION/Au/NPs were dispersed using ultrasonic (USC-T, VWR, Radnor, USA) 40 W for 10 min. The prepared NPs (SPIONs/Au/NPs) were subsequently modified with 438 µL 1% acetic acid and a 62 µL of CS (8 mg/mL). Incubation of the NPs took place for 30 min on a rotator (Multi-RS 60, Biosan, Riga, Latvia) (rpm 30; deg. 84°/10; vibro pause 5°/5). To create the CS polymer structure, 500 µL TPP (0.1 mg/mL in 1% acetic acid) was added. Subsequently, NPs were incubated on a rotator (Multi-RS 60, Biosan, Riga, Latvia) for 30 min (rpm 30; deg. 84°/10; vibro pause 5°/5) and temperature of 22 °C to produce SPIONs/Au/CS/NPs. SPIONs/Au/CS/NPs (40 mg) were washed 3 times with 1000 µL of 1% acetic acid, 120 rpm for 60 s, and then separated using a neodymium magnet and stored at 4 °C for further use.

### 4.4. Modification of SPIONs/Au/CS/NPs with Sarcosine Oxidase (SOX)

SPIONs/Au/NPs (40 mg) were washed 3 times with 1000 µL of 1× PBS (pH 7.0), 120 rpm for 60 s, and then separated using a neodymium magnet. Subsequently, SPIONs/Au/NPs were dispersed using ultrasonic (USC-T, VWR, Radnor, USA) 40 W for 10 min. The prepared NPs (SPIONs/Au/NPs) were then modified with 338 µL of 1% acetic acid and 62 µL of CS (8 mg/mL) and 100 µL of SOX. Incubation of the NPs took place for 30 min on a rotator (Multi-Rotator RS 60, Biosan, Riga, Latvia) (rpm 30; deg. 84°/10; vibro pause 5°/5). To form the CS polymer structure, 500 μL of TPP (0.1 mg/mL in 1% acetic acid) was added. Subsequently, NPs were incubated on a rotator (Multi-Rotator RS 60, Biosan, Riga, Latvia) for 30 min (rpm 30; deg. 84°/10; vibro pause 5°/5) and temperature of 22 °C to produce SPIONs/Au/CS/SOX/NPs. SPIONs/Au/CS/SOX/NPs (40 mg) were washed 3 times with 1000 µL of 1% acetic acid, 120 rpm for 60 s, and then separated using a neodymium magnet and stored at 4 °C for further use.

### 4.5. Scanning Electron Microscopy

Structure of NPs was characterized by scanning electron microscopy (SEM). For documentation of the NPs structure, the MIRA3 LMU (Tescan, Brno, Czech Republic) was used. This model is equipped with a high brightness Schottky field emitter for low noise imaging at fast scanning rates. The SEM was fitted with In-Beam SE detector. An accelerating voltage of 15 kV and beam currents about 1 nA gave satisfactory results regarding maximum throughput.

### 4.6. Absorbance Measurements

Absorbance scan was carried out in the range 300–850 nm by 2 nm steps. The samples for measurements (100 µL) were placed in 96-well UV plate (IAB, Prague, Czech Republic). All measurements were performed at 22 °C (V UV-3100PC, VWR, Radnor, USA). The absorbance or spectra in plate (Brand, Wertheim, Germany) were recorded by using a reader Infinite M200 (Tecan, Männedorf, Switzerland).

### 4.7. Zetasizer Analysis of Nanoparticles

The size distribution (i.e., the hydrodynamic diameter, DH) was determined by dynamic light scattering (DLS) using the Zetasizer Nano ZS ZEN3600 (Malvern Instruments, Malvern, UK) with the detection angle of 173° in optically homogeneous square polystyrene cells. The samples were diluted hundredfold with deionized water. All measurements were performed at 25 °C. Each value was obtained as an average of 5 runs with at least 10 measurements. Version 7.10 of the Zetasizer Software was applied for data evaluation. The particle charge (ζ-potential) was measured by the microelectrophoretic method using a Malvern Zetasizer Nano ZS ZEN3600 (Malvern Instruments, Malvern, UK). All the measurements were performed at 25 °C in polycarbonate cuvettes. Each value was obtained as an average of 5 subsequent runs of the instrument with at least 20 measurements.

### 4.8. Ninhydrin Assay for Chitosan (CS) Detection

The reaction solutions were then processed as described by Sabnis and Block [139] with a modification reported in our previous study [109]. The ninhydrin reagent was freshly prepared on the day of the assay by adding 25 mL of 4 M acetate buffer (pH 5.2) to 2 g ninhydrin and 0.3 g hydrindantin in 75 mL DMSO. For the assay, 75 µL of reagent was added to 100 µL of the sample in an Eppendorf microtube. The microtubes were immediately capped, briefly shaken by hand and heated in a thermoblock at 99 °C for 30 min to allow the reaction to proceed. After cooling, 15 mL of a 1:1 ethanol: water mixture was added to each sample. The color intensity of the complex was spectrophotometrically evaluated as a measure of depolymerized CS activity. Accurately weighed depolymerized CSs were dissolved in 1% *w*/*v* acetic acid. A blank solution was also prepared in an identical manner, wherein 1% *w*/*v* acetic acid was used instead of a CS solution to prepare the reaction mixture. The effect of the solvent system was nullified by calibrating the instrument to 100% transmittance of the blank. Particles preparation (SPIONs/Au/CS/SOX/NPs): 25 μL of NPs and 145 μL of ninhydrin reagent were pipetted. Then, incubation on a thermoblock (Bio-DTB-100, Biosan, Riga, Latvia) at 100 °C for 30 min was carried out. After incubation, the solution was pipetted into the plate, free of particles, which were held by the magnet. Measurement was performed by using a reader Infinite M200 (Tecan, Männedorf, Switzerland): 250–800 nm, at 26 °C, step 2 nm. Relative absorbance (RA ninhydrin factor) of each prepared SPIONs solution was calculated as follows:

RA = absorbance of 100 µg/mL CS solution/absorbance of NPs solution(1)

### 4.9. SOX Activity of SPIONs/Au/CS/SOX/NPs

For SOX activity of SPIONs/Au/CS/SOX/NPs measurement, the concentration of SPIONs of 40 mg/mL was used. For reaction with the sample (sarcosine), 0.2 M phosphate buffer (pH 8), 4-aminoantipyrine (0.1–5 mM), and phenol (1–25 mM) were used. The ratio of the sample to the reaction solution was 1:5. Subsequently, the samples were shaken every second minute for 30 min (3 s, 5 amplitudes). After 30 min, the particles were separated, the solution was pipetted into a new well of the microtiter plate. Measurement was performed at a wavelength of 500 nm at laboratory temperature.

### 4.10. Pseudo-Peroxidase Assay

NPs (10 µL) were pipetted into plate (Brand, Wertheim, Germany) to 200 µL of substrate solution. The substrate solution was composed of: 915 µL of 0.5 M acetate buffer (pH 4.0) and 100 µL of 5 mM TMB (100% DMSO) along with 85 µL H_2_O_2_ (30%). CS-modified SPIONs were first washed (3 times at 120 rpm for 10 s, and then separated using a neodymium magnet) with 200 mM phosphate buffer (100 μL) at pH 8. Washing the modified SPIONs was carried out in a well of the microtiter plate where 200 μL of 200 mM phosphate buffer (pH 8.0) was added to 10 μL of particles, then the particles were separated by a magnet. Subsequently, 200 μL of substrate solution with TMB was added to the particles which remained in the wall. After 30 min incubation, with shaking at 2 min intervals, the color development appeared. After another 30 min, the particles were separated and the reacted solution was pipetted into a new plate and measured at 650 nm. The color development appeared after incubation. The absorbance or spectra were recorded by using a reader Infinite M200 (Tecan, Männedorf, Switzerland). Spectra are very well observable at 650 nm. The measurement parameters were: 2 nm step, temperature 25 °C, and maximum 650 nm. To evaluate the pseudo-peroxidase activity, the absorbance value was used at 30 min. As a control sample for assessment of pseudo-peroxidase activity, 10 µL of 1 U horseradish peroxidase (HRP) and 200 µL of substrate solution was used per reaction.

### 4.11. Creatinine Analysis

Creatinine was determined using Greiner procedures on BS-200 Chemical Analyser (Mindray, Shenzhen, China). The method for the analysis of creatinine was evaluated at 510 nm (picrate method [140]). The recording of experimental data was performed as a kinetic reaction at 16 s-intervals period. Parameters of the test were: incubation time 1 min and reaction time 2 min. The amount of pipetted reagent R1 (0.16 mM sodium hydrochloride) was 180 μL, and R2 (4 mM, picric acid) 30 μL. 10 μL of urine (20 times diluted with distilled water) was used in the reaction. Concentrations of creatinine were determined according to the following equation:

*y* = 1.5266*x* + 3.1532, *r* = 0.9994 (2)

### 4.12. Growth Inhibition Assay

*Saccharomyces cerevisiae* (ATCC 9763), *Escherichia coli* and *Staphylococcus aureus* were obtained from the Czech Collection of Microorganisms, Faculty of Science, Masaryk University, Brno, Czech Republic. For the determination of growth curves of experimental models selected, the LB medium (trypton, yeast extract, NaCl) was used. The cultures were first cultivated for 24 h, then diluted to 0.1 of the optical density (OD) at 600 nm and placed in a well for 24 h. Cultivated yeast (*S. cerevisiae*) and microorganisms (*E. coli*, *S. aureus*) were diluted using LB media to the OD of 0.1 before measurement. 250 µL of diluted yeast or microorganisms was pipetted into each well and 50 µL of the subsequent tested sample was added. As a control, the 300 µL of LB media was added to the separate well. The measurement was carried out on the Infinite 200 PRO multifunctional microplate reader (Tecan, Männedorf, Switzerland) at 600 nm for 18 h and 37 °C. The absorbance value was recorded every 30 min. Each sample was analysed using 5 independent repetitions. The results were expressed as a average value. After measuring the growth curves, the IC_50_ was calculated by probit analysis using the QINSLAB information system.

### 4.13. Hemolytic Assay

Hemolytic assay was done on erythrocytes. Plasma from the fresh blood sample (RBC 5.16 ∙ 10^12^ L; HBG 161 g/L; MCV 83.6 fL, hematology analyzer Mindray BC-5500, Shenzhen, China) was removed by multiple washings with PBS (35 mM phosphate, pH 7.0, 150 mM NaCl) and re-suspended to 4% (*v*/*v*) in PBS. Then, 0.2 mL of washed blood and 0.8 mL of NPs (NPs were diluted in PBS) were mixed in an Eppendorf microtube, and the mixture was incubated for 2 h, with gentle stirring at half hour intervals. After incubation, the mixture was centrifugated at 2000 g for 10 min. Then, 100 μL of the supernatant was taken into the microtiter plate. Absorbance at 570 nm and absorption spectrum from 300 to 850 nm were measured. As a positive and a negative control, 0.8 mL of 18 MΩ water was mixed with 0.2 mL of RBC, and 0.8 mL of PBS with 0.2 mL of RBC, respectively. After completion of the incubation period, the cells were centrifuged, and the absorbance of the supernatant containing lysed erythrocytes was measured at 570 nm. The percentage of the hemolysis was determined by the following equation:

% hemolysis = [(A_t_ − A_c_)/(A_100%_ − A_c_)] × 100 (3)

where A_t_ is the absorbance of the supernatant from samples incubated with the particles, A_c_ is the absorbance of the supernatant from negative control (PBS), and A_100%_ is the absorbance of the positive control supernatant.

### 4.14. Biological Samples of Urine

Urine samples obtained from healthy volunteers (K) were collected at time intervals. Samples P were obtained from patients-voluntary donors with a histologically confirmed diagnosis of PCa. All procedures were performed in accordance with ethical principles. Sarcosine content was analyzed by the proposed method. All participants signed informed consent to use their biological sample (E01/2017). From all participants, the informed consent for the collection and analysis of clinical samples was obtained. Sample analysis was performed by the methodology developed in this article.

### 4.15. Data Treatment and Descriptive Statistics

The experimental work was carried out in 3 independent experiments. The analysis of each sample was carried out 5 times. The obtained data are presented as average values. From the proposed study no experimental subjects were excluded from the proposed experimental studies. All the obtained data were stored in the QINSLAB database. If possible, data were processed and evaluated mathematically and statistically in the QINSLAB database. Photographs were processed by programme ColorTest, which assigns intensity to the individual pixels of the studied image in the color area. For publication purposes, data were processed using Microsoft (Redmond, WA, USA).

## 5. Conclusions

The role of sarcosine is substantial in both physiological and pathophysiological processes. The amount of sarcosine can be bioanalytically determined by liquid chromatography, but this method does not achieve the required sensitivity. Therefore, new ways for sarcosine determination are sought. In this work, we used nanoconstructs—SPIONs—to develop a biosensor for sarcosine as a potential biomarker for prostate cancer. To increase the sensitivity of the assay, pseudo-peroxidase activity of gold SPIONs was utilized. The designed detection system was tested in artificial urine specimens and also applied to real urine specimens. The proposed procedure enables the analysis of sarcosine at micromolar levels, and is very sensitive, affordable, and suitable for monitoring urine sarcosine concentrations in prostate cancer patients, and could also be used for screening testing. The proposed technology allows subsequent development in the field of designing new types of fluid chips or microchips for targeted personalized medicine.

## Figures and Tables

**Table 1:** Analytical description of the determination of sarcosine in matrices.

Substance	Regresion Equation	Linear Dynamic Range (µM)	Correlation Coefficient (r)	LOD (µM)	LOQ (µM)	RSD (%)
Sarcosine ^#^	*y* = 0.0011*x* − 0.0001	17–100	0.9992	5	17	6.35
Sarcosine *	*y* = 0.0010*x* + 0.0006	59–100	0.9912	18	59	21.35

^#^ (in water); * (in artifical urine); five repetitions; limit of detection (LOD) and limit of quantification (LOQ) were calculated by ISO (International Organization for Standardization) method.

## Abbreviations

*r*	correlation coefficient
LOD	limit of detection determined by the ISO method
LOQ	limit of detection determined by the second ISO method
RSD	relative standard deviation calculated from the individual measurements and fit into the straight line equation and compared to the input value
SOX	sarcosine oxidase (EC 1.5.3.1)
SPION	superparamagnetic iron oxide nanoparticles
TPP	sodium tripolyphosphate
TMB	3,3′,5,5′-tetramethylbenzidine
CS	chitosan
4-AAP	4-aminoantipyrine
IC_50_	the half maximal inhibitory concentration
RBC	red blood cell
AuNPs	gold nanoparticles

## References

[1] Bar-Joseph I., Pras E., Reznik-Wolf H., Marek-Yagel D., Abu-Horvitz A., Dushnitzky M., Goldstein N., Rienstein S., Dekel M., Pode-Shakked B. (2012). Mutations in the sarcosine dehydrogenase gene in patients with sarcosinemia. Hum. Genet..

[2] Scriver C.R. (2001). The metabolic & molecular bases of inherited disease.

[3] Sreekumar A., Poisson L.M., Rajendiran T.M., Khan A.P., Cao Q., Yu J., Laxman B., Mehra R., Lonigro R.J., Li Y. (2009). Metabolomic profiles delineate potential role for sarcosine in prostate cancer progression. Nature.

[4] Allen R.H., Stabler S.P., Lindenbaum J. (1993). Serum betaine, *N*, *N*-dimethylglycine and *N*-methylglycine levels in patients with cobalamin and folate deficiency and related inborn errors of metabolism. Metabolism.

[5] Steenkamp D.J., Husain M. (1982). The effect of tetrahydrofolate on the reduction of electron transfer flavoprotein by sarcosine and dimethylglycine dehydrogenases. Biochem. J..

[6] Wang L., Liu S., Yang W., Yu H., Zhang L., Ma P., Wu P., Li X., Cho K., Xue S. (2017). Plasma amino acid profile in patients with aortic dissection. Sci. Rep..

[7] Munshi S.U., Rewari B.B., Bhavesh N.S., Jameel S. (2013). Nuclear magnetic resonance based profiling of biofluids reveals metabolic dysregulation in HIV-infected persons and those on anti-retroviral therapy. PLoS ONE.

[8] Lane H.-Y., Lin C.-H., Huang Y.-J., Liao C.-H., Chang Y.-C., Tsai G.E. (2010). A randomized, double-blind, placebo-controlled comparison study of sarcosine (*N*-methylglycine) and *D*-serine add-on treatment for schizophrenia. Int. J. Neuropsychopharmacol..

[9] Tsai G., Lane H.-Y., Yang P., Chong M.-Y., Lange N. (2004). Glycine transporter I inhibitor, *N*-methylglycine (sarcosine), added to antipsychotics for the treatment of schizophrenia. Biol. Psychiatry.

[10] McKeever M., Weir D., Molloy A., Scott J. (1991). Betaine-homocysteine methyltransferase: Organ distribution in man, pig and rat and subcellular distribution in the rat. Clin. Sci..

[11] Schwahn B.C., Hafner D., Hohlfeld T., Balkenhol N., Laryea M.D., Wendel U. (2003). Pharmacokinetics of oral betaine in healthy subjects and patients with homocystinuria. Br. J. Clin. Pharmcol..

[12] Benevenga N.J. (2007). Consideration of betaine and one-carbon sources of *N*^5^-methyltetrahydrofolate for use in homocystinuria and neural tube defects. Am. J. Clin. Nutr..

[13] Singh R.H., Kruger W.D., Wang L., Pasquali M., Elsas II L.J. (2004). Cystathionine β-synthase deficiency: Effects of betaine supplementation after methionine restriction in B6-nonresponsive homocystinuria. Genet. Med..

[14] Ferlay J., Soerjomataram I., Dikshit R., Eser S., Mathers C., Rebelo M., Parkin D.M., Forman D., Bray F. (2015). Cancer incidence and mortality worldwide: Sources, methods and major patterns in GLOBOCAN 2012. Int. J. Cancer.

[15] Edwards D.R., Moroz K., Zhang H., Mulholland D., Abdel-Mageed A.B., Mondal D. (2018). PRL-3 increases the aggressive phenotype of prostate cancer cells in vitro and its expression correlates with high-grade prostate tumors in patients. Int. J. Oncol..

[16] Pihikova D., Pakanova Z., Nemcovic M., Barath P., Belicky S., Bertok T., Kasak P., Mucha J., Tkac J. (2016). Sweet characterisation of prostate specific antigen using electrochemical lectin-based immunosensor assay and MALDI TOF/TOF analysis: Focus on sialic acid. Proteomics.

[17] Hayes J.H., Barry M.J. (2014). Screening for prostate cancer with the prostate-specific antigen test: A review of current evidence. JAMA.

[18] De Kok J.B., Verhaegh G.W., Roelofs R.W., Hessels D., Kiemeney L.A., Aalders T.W., Swinkels D.W., Schalken J.A. (2002). DD3PCA3, a very sensitive and specific marker to detect prostate tumors. Cancer Res..

[19] Font-Tello A., Juanpere N., de Muga S., Lorenzo M., Lorente J.A., Fumado L., Serrano L., Serrano S., Lloreta J., Hernández S. (2015). Association of ERG and TMPRSS2-ERG with grade, stage, and prognosis of prostate cancer is dependent on their expression levels. Prostate.

[20] Hagiwara K., Tobisawa Y., Kaya T., Kaneko T., Hatakeyama S., Mori K., Hashimoto Y., Koie T., Suda Y., Ohyama C. (2017). Wisteria Floribunda Agglutinin and its Reactive-Glycan-Carrying Prostate-Specific Antigen as a novel diagnostic and prognostic marker of prostate cancer. Int. J. Mol. Sci..

[21] Hessels D., Gunnewiek J.M.K., van Oort I., Karthaus H.F., van Leenders G.J., van Balken B., Kiemeney L.A., Witjes J.A., Schalken J.A. (2003). DD3PCA3-based molecular urine analysis for the diagnosis of prostate cancer. Eur. Urol..

[22] Kulkarni P., Uversky V.N. (2017). Cancer/Testis Antigens:“Smart” biomarkers for diagnosis and prognosis of prostate and other cancers. Int. J. Mol. Sci..

[23] Nakanishi H., Groskopf J., Fritsche H.A., Bhadkamkar V., Blase A., Kumar S.V., Davis J.W., Troncoso P., Rittenhouse H., Babaian R.J. (2008). PCA3 molecular urine assay correlates with prostate cancer tumor volume: Implication in selecting candidates for active surveillance. J. Urol..

[24] Tomlins S.A., Laxman B., Varambally S., Cao X., Yu J., Helgeson B.E., Cao Q., Prensner J.R., Rubin M.A., Shah R.B. (2008). Role of the TMPRSS2-ERG gene fusion in prostate cancer. Neoplasia.

[25] Tseng T.-L., Shih Y.-P., Huang Y.-C., Wang C.-K., Chen P.-H., Chang J.-G., Yeh K.-T., Chen Y.-M.A., Buetow K.H. (2003). Genotypic and phenotypic characterization of a putative tumor susceptibility gene, *GNMT*, in liver cancer. Cancer Res..

[26] Huang Y.-C., Lee C.-M., Chen M., Chung M.-Y., Chang Y.-H., Huang W.J.-S., Ho D.M.-T., Pan C.-C., Wu T.T., Yang S. (2007). Haplotypes, loss of heterozygosity, and expression levels of glycine *N*-methyltransferase in prostate cancer. Clin. Cancer Res..

[27] Hatziapostolou M., Iliopoulos D. (2011). Epigenetic aberrations during oncogenesis. Cell. Mol. Life Sci..

[28] Kelavkar U., Harya N., Hutzley J., Bacich D., Monzon F., Chandran U., Dhir R., O’Keefe D. (2007). DNA methylation paradigm shift: 15-lipoxygenase-1 upregulation in prostatic intraepithelial neoplasia and prostate cancer by atypical promoter hypermethylation. Prostagl. Lipid Med..

[29] Pierconti F., Martini M., Pinto F., Cenci T., Capodimonti S., Calarco A., Bassi P.F., Larocca L.M. (2011). Epigenetic silencing of SOCS3 identifies a subset of prostate cancer with an aggressive behavior. Prostate.

[30] Ianni M., Porcellini E., Carbone I., Potenzoni M., Pieri A., Pastizzaro C., Benecchi L., Licastro F. (2013). Genetic factors regulating inflammation and DNA methylation associated with prostate cancer. Prost. Cancer Prost. Dis..

[31] Luka Z., Mudd S.H., Wagner C. (2009). Glycine *N*-methyltransferase and regulation of *S*-adenosylmethionine levels. J. Biol. Chem..

[32] Khan A.P., Rajendiran T.M., Bushra A., Asangani I.A., Athanikar J.N., Yocum A.K., Mehra R., Siddiqui J., Palapattu G., Wei J.T. (2013). The role of sarcosine metabolism in prostate cancer progression. Neoplasia.

[33] Baronia R., Singh M., Gupta R.B., Karuppiah S., Kumar R., Belz J., Shanker R., Sridhar S., Singh S.P. (2018). Synthesis and characterization of multifunctional gold nanoclusters for application in radiation therapy. Int. J. Nanomed..

[34] De Boer B., Kahlman J., Jansen T., Duric H., Veen J. (2007). An integrated and sensitive detection platform for magneto-resistive biosensors. Biosens. Bioelectron..

[35] Cai S., Qi C., Li Y., Han Q., Yang R., Wang C. (2016). PtCo bimetallic nanoparticles with high oxidase-like catalytic activity and their applications for magnetic-enhanced colorimetric biosensing. J. Mater. Chem. B.

[36] Patel P., Kansara K., Singh R., Shukla R.K., Singh S., Dhawan A., Kumar A. (2018). Cellular internalization and antioxidant activity of cerium oxide nanoparticles in human monocytic leukemia cells. Int. J. Nanomed..

[37] Purohit R., Singh S. (2018). Fluorescent gold nanoclusters for efficient cancer cell targeting. Int. J. Nanomed..

[38] Siow W.X., Chang Y.-T., Babič M., Lu Y.-C., Horák D., Ma Y.-H. (2018). Interaction of poly-*L*-lysine coating and heparan sulfate proteoglycan on magnetic nanoparticle uptake by tumor cells. Int. J. Nanomed..

[39] Unterweger H., Dézsi L., Matuszak J., Janko C., Poettler M., Jordan J., Bäuerle T., Szebeni J., Fey T., Boccaccini A.R. (2018). Dextran-coated superparamagnetic iron oxide nanoparticles for magnetic resonance imaging: Evaluation of size-dependent imaging properties, storage stability and safety. Int. J. Nanomed..

[40] Yang Z., Duan J., Wang J., Liu Q., Shang R., Yang X., Lu P., Xia C., Wang L., Dou K. (2018). superparamagnetic iron oxide nanoparticles modified with polyethylenimine and galactose for siRNA targeted delivery in hepatocellular carcinoma therapy. Int. J. Nanomed..

[41] Zhang Q., Li L., Qiao Z., Lei C., Fu Y., Xie Q., Yao S., Li Y., Ying Y. (2017). Electrochemical Conversion of Fe3O4 Magnetic Nanoparticles to Electroactive Prussian Blue Analogues for Self-Sacrificial Label Biosensing of Avian Influenza Virus H5N1. Anal. Chem..

[42] Haun J.B., Yoon T.J., Lee H., Weissleder R. (2010). Magnetic nanoparticle biosensors. Wiley Interdiscip. Rev..

[43] Jia Y., Peng Y., Bai J., Zhang X., Cui Y., Ning B., Cui J., Gao Z. (2018). Magnetic nanoparticle enhanced surface plasmon resonance sensor for estradiol analysis. Sens. Actuators B Chem..

[44] Pakapongpan S., Poo-Arporn R.P. (2017). Self-assembly of glucose oxidase on reduced graphene oxide-magnetic nanoparticles nanocomposite-based direct electrochemistry for reagentless glucose biosensor. Mater. Sci. Eng..

[45] Ye W., Ding Y., Sun Y., Tian F., Yang M. (2017). A Nanoporous Alumina Membrane Based Impedance Biosensor for Histamine Detection with Magnetic Nanoparticles Separation and Amplification. Proc. Technol..

[46] Zhao J., Dong W., Zhang X., Chai H., Huang Y. (2018). FeNPs@ Co3O4 hollow nanocages hybrids as effective peroxidase mimics for glucose biosensing. Sens. Actuators B Chem..

[47] Yuan L.-F., He Y.-J., Zhao H., Zhou Y., Gu P. (2014). Colorimetric detection of *D*-amino acids based on anti-aggregation of gold nanoparticles. Chin. Chem. Lett..

[48] Zhang F.X., Han L., Israel L.B., Daras J.G., Maye M.M., Ly N.K., Zhong C.-J. (2002). Colorimetric detection of thiol-containing amino acids using gold nanoparticles. Analyst.

[49] Zhang Q., Zhang D., Lu Y., Xu G., Yao Y., Li S., Liu Q. (2016). Label-free amino acid detection based on nanocomposites of graphene oxide hybridized with gold nanoparticles. Biosens. Bioelectron..

[50] Nikitin M., Orlov A., Znoyko S., Bragina V., Gorshkov B., Ksenevich T., Cherkasov V., Nikitin P. (2017). Multiplex biosensing with highly sensitive magnetic nanoparticle quantification method. J. Magn. Magn. Mater..

[51] Tamanaha C., Mulvaney S., Rife J., Whitman L. (2008). Magnetic labeling, detection, and system integration. Biosens. Bioelectron..

[52] Xu T., Chi B., Wu F., Ma S., Zhan S., Yi M., Xu H., Mao C. (2017). A sensitive label-free immunosensor for detection α-Fetoprotein in whole blood based on anticoagulating magnetic nanoparticles. Biosens. Bioelectron..

[53] Heger Z., Cernei N., Krizkova S., Masarik M., Kopel P., Hodek P., Zitka O., Adam V., Kizek R. (2015). Paramagnetic nanoparticles as a platform for FRET-based sarcosine picomolar detection. Sci. Rep..

[54] Pamme N. (2012). On-chip bioanalysis with magnetic particles. Curr. Opin. Chem. Biol..

[55] Adam V., Huska D., Hubalek J., Kizek R. (2010). Easy to use and rapid isolation and detection of a viral nucleic acid by using paramagnetic microparticles and carbon nanotubes-based screen-printed electrodes. Microfluid. Nanofluid..

[56] Ng A.H., Choi K., Luoma R.P., Robinson J.M., Wheeler A.R. (2012). Digital microfluidic magnetic separation for particle-based immunoassays. Anal. Chem..

[57] Kim S., Kim D., Kim S. (2018). A rapid real-time quantification in hybrid paper-polymer centrifugal optical devices. Biosens. Bioelectron..

[58] Trinder P. (1969). Determination of glucose in blood using glucose oxidase with an alternative oxygen acceptor. Ann. Clin. Biochem..

[59] Wiewiorka O., Dastych M., Cermakova Z. (2017). Trinder Reaction in Clinical Biochemistry-Benefits and Limits. Chem. Listy.

[60] Yamkamon V., Phakdee B., Yainoy S., Suksrichawalit T., Tatanandana T., Sangkum P., Eiamphungporn W. (2018). Development of sarcosine quantification in urine based on enzyme-coupled colorimetric method for prostate cancer diagnosis. EXCLI J..

[61] Cernei N., Zitka O., Skalickova S., Gumulec J., Masarik M., Hrabec R., Adam V., Kizek R. (2012). Sarcosine in urine of patients with prostate carcinome. Prakt. Lek..

[62] El-Boubbou K., Gruden C., Huang X. (2007). Magnetic glyco-nanoparticles: A unique tool for rapid pathogen detection, decontamination, and strain differentiation. J. Am. Chem. Soc..

[63] Guo Q., Teng X., Rahman S., Yang H. (2003). Patterned Langmuir—Blodgett films of monodisperse nanoparticles of iron oxide using soft lithography. J. Am. Chem. Soc..

[64] Haviv A.H., Grenèche J.-M., Lellouche J.-P. (2010). Aggregation control of hydrophilic maghemite (γ-Fe_2_O_3_) nanoparticles by surface doping using cerium atoms. J. Am. Chem. Soc..

[65] Scialabba C., Licciardi M., Mauro N., Rocco F., Ceruti M., Giammona G. (2014). Inulin-based polymer coated SPIONs as potential drug delivery systems for targeted cancer therapy. Eur. J. Pharm. Biopharm..

[66] Inamdar N.N., Mourya V. (2014). Chitosan and Low Molecular Weight Chitosan: Biological and Biomedical Applications. Adv. Biomater. Biodev..

[67] Kumar P., Jaiwal R., Pundir C. (2017). An improved amperometric creatinine biosensor based on nanoparticles of creatininase, creatinase and sarcosine oxidase. Anal. Biochem..

[68] Narwal V., Kumar P., Joon P., Pundir C. (2018). Fabrication of an amperometric sarcosine biosensor based on sarcosine oxidase/chitosan/CuNPs/c-MWCNT/Au electrode for detection of prostate cancer. Enzyme Microbiol. Technol..

[69] Lad U., Kale G.M., Bryaskova R. (2014). Sarcosine oxidase encapsulated polyvinyl alcohol-silica-AuNP hybrid films for sarcosine sensing electrochemical bioelectrode. J. Electrochem. Soc..

[70] Nguy T.P., van Phi T., Tram D.T., Eersels K., Wagner P., Lien T.T. (2017). Development of an impedimetric sensor for the label-free detection of the amino acid sarcosine with molecularly imprinted polymer receptors. Sens. Actuators B Chem..

[71] Hirsch V., Kinnear C., Moniatte M., Rothen-Rutishauser B., Clift M.J., Fink A. (2013). Surface charge of polymer coated SPIONs influences the serum protein adsorption, colloidal stability and subsequent cell interaction in vitro. Nanoscale.

[72] Park J., Yu M.K., Jeong Y.Y., Kim J.W., Lee K., Phan V.N., Jon S. (2009). Antibiofouling amphiphilic polymer-coated superparamagnetic iron oxide nanoparticles: Synthesis, characterization, and use in cancer imaging in vivo. J. Mater. Chem..

[73] Mardinoglu A., Cregg P. (2015). Modelling the effect of SPION size in a stent assisted magnetic drug targeting system with interparticle interactions. Sci. World J..

[74] Kapri A., Zaidi M., Satlewal A., Goel R. (2010). SPION-accelerated biodegradation of low-density polyethylene by indigenous microbial consortium. Int. Biodeter. Biodegrad..

[75] Herve K., Douziech-Eyrolles L., Munnier E., Cohen-Jonathan S., Souce M., Marchais H., Limelette P., Warmont F., Saboungi M., Dubois P. (2008). The development of stable aqueous suspensions of PEGylated SPIONs for biomedical applications. Nanotechnology.

[76] Xie J., Xu C., Kohler N., Hou Y., Sun S. (2007). Controlled PEGylation of monodisperse Fe_3_O_4_ nanoparticles for reduced non-specific uptake by macrophage cells. Adv. Mater..

[77] HUANG Z.r. (2008). Development and evaluation of lipid nanoparticles for camptothecin delivery: A comparison of solid lipid nanoparticles, nanostructured lipid carriers, and lipid emulsion. Acta Pharmacolog. Sinica.

[78] Müller R.H., MaÈder K., Gohla S. (2000). Solid lipid nanoparticles (SLN) for controlled drug delivery—A review of the state of the art. Eur. J. Pharm. Biopharm..

[79] Mahmoudi M., Sant S., Wang B., Laurent S., Sen T. (2011). Superparamagnetic iron oxide nanoparticles (SPIONs): Development, surface modification and applications in chemotherapy. Adv. Drug Dev. Rev..

[80] Dubey S.P., Lahtinen M., Sillanpää M. (2010). Green synthesis and characterizations of silver and gold nanoparticles using leaf extract of Rosa rugosa. Colloids Surf. A.

[81] Dubey S.P., Lahtinen M., Sillanpää M. (2010). Tansy fruit mediated greener synthesis of silver and gold nanoparticles. Process Biochem..

[82] Sonavane G., Tomoda K., Makino K. (2008). Biodistribution of colloidal gold nanoparticles after intravenous administration: Effect of particle size. Colloids Surf. B.

[83] De Palma R., Peeters S., van Bael M.J., van den Rul H., Bonroy K., Laureyn W., Mullens J., Borghs G., Maes G. (2007). Silane ligand exchange to make hydrophobic superparamagnetic nanoparticles water-dispersible. Chem. Mater..

[84] Shukla A., Mishra V., Bhoop B.S., Katare O.P. (2015). Alginate coated chitosan microparticles mediated oral delivery of diphtheria toxoid.(Part A). Systematic optimization, development and characterization. Int. J. Pharm..

[85] Burda C., Chen X., Narayanan R., El-Sayed M.A. (2005). Chemistry and properties of nanocrystals of different shapes. Chemical Rev..

[86] He W., Wamer W., Xia Q., Yin J.-j., Fu P.P. (2014). Enzyme-like activity of nanomaterials. J. Environ. Sci. Health.

[87] Dai Z., Liu S., Bao J., Ju H. (2009). Nanostructured FeS as a mimic peroxidase for biocatalysis and biosensing. Chem. A Eur. J..

[88] Gao L., Zhuang J., Nie L., Zhang J., Zhang Y., Gu N., Wang T., Feng J., Yang D., Perrett S. (2007). Intrinsic peroxidase-like activity of ferromagnetic nanoparticles. Nat. Nanotechnol..

[89] He W., Wu X., Liu J., Hu X., Zhang K., Hou S., Zhou W., Xie S. (2010). Design of AgM bimetallic alloy nanostructures (M = Au, Pd, Pt) with tunable morphology and peroxidase-like activity. Chem. Mater..

[90] Jv Y., Li B., Cao R. (2010). Positively-charged gold nanoparticles as peroxidiase mimic and their application in hydrogen peroxide and glucose detection. Chem. Commun..

[91] Luo W., Li Y.-S., Yuan J., Zhu L., Liu Z., Tang H., Liu S. (2010). Ultrasensitive fluorometric determination of hydrogen peroxide and glucose by using multiferroic BiFeO3 nanoparticles as a catalyst. Talanta.

[92] Song Y., Wang X., Zhao C., Qu K., Ren J., Qu X. (2010). Label-free colorimetric detection of single nucleotide polymorphism by using single-walled carbon nanotube intrinsic peroxidase-like activity. Chem. A Eur. J..

[93] Wei H., Wang E. (2008). Fe_3_O_4_ magnetic nanoparticles as peroxidase mimetics and their applications in H_2_O_2_ and glucose detection. Anal. Chem..

[94] Wu L.-L., Wang L.-Y., Xie Z.-J., Xue F., Peng C.-F. (2016). Colorimetric detection of Hg^2+^ based on inhibiting the peroxidase-like activity of DNA–Ag/Pt nanoclusters. RSC Adv..

[95] Zhang Z., Wang Z., Wang X., Yang X. (2010). Magnetic nanoparticle-linked colorimetric aptasensor for the detection of thrombin. Sens. Actuators B Chem..

[96] Asati A., Santra S., Kaittanis C., Nath S., Perez J.M. (2009). Oxidase-like activity of polymer-coated cerium oxide nanoparticles. Angew. Chem. Int. Ed..

[97] Jiang C., Zhu J., Li Z., Luo J., Wang J., Sun Y. (2017). Chitosan–gold nanoparticles as peroxidase mimic and their application in glucose detection in serum. RSC Advances.

[98] Wang X., Hu Y., Wei H. (2016). Nanozymes in bionanotechnology: From sensing to therapeutics and beyond. Inorg. Chem. Front..

[99] Shah J., Purohit R., Singh R., Karakoti A.S., Singh S. (2015). ATP-enhanced peroxidase-like activity of gold nanoparticles. J. Colloid Interface Sci..

[100] Xu J., Zeng F., Wu S., Liu X., Hou C., Tong Z. (2007). Gold nanoparticles bound on microgel particles and their application as an enzyme support. Nanotechnology.

[101] Lan D., Li B., Zhang Z. (2008). Chemiluminescence flow biosensor for glucose based on gold nanoparticle-enhanced activities of glucose oxidase and horseradish peroxidase. Bios. Bioelectron..

[102] Duan D., Fan K., Zhang D., Tan S., Liang M., Liu Y., Zhang J., Zhang P., Liu W., Qiu X. (2015). Nanozyme-strip for rapid local diagnosis of Ebola. Biosens. Bioelectron..

[103] Fang H., Pan Y., Shan W., Guo M., Nie Z., Huang Y., Yao S. (2014). Enhanced nonenzymatic sensing of hydrogen peroxide released from living cells based on Fe_3_ O_4_/self-reduced graphene nanocomposites. Anal. Methods.

[104] Wong A.C., Wright D.W., Conrad J.A. (2015). Functionalized gold nanoparticles for detection of cancer biomarkers. General Methods in Biomarker Research and their Applications.

[105] Zurek M., Kremplova M., Nejdl L., Hynek D., Kopel P., Adam V., Kizek R. (2014). Characterization of gold nanoparticles-modified CdTe quantum dots by scanning electrochemical microscopy. NANOCON.

[106] Chudobova D., Cihalova K., Skalickova S., Zitka J., Rodrigo M.A.M., Milosavljevic V., Hynek D., Kopel P., Vesely R., Adam V. (2015). 3D-printed chip for detection of methicillin-resistant *Staphylococcus aureus* labeled with gold nanoparticles. Electrophoresis.

[107] Marquez L.A., Dunford H.B. (1997). Mechanism of the oxidation of 3,5,3′,5′-tetramethylbenzidine by myeloperoxidase determined by transient-and steady-state kinetics. Biochemistry.

[108] Lv X., Weng J. (2013). Ternary composite of hemin, gold nanoparticles and graphene for highly efficient decomposition of hydrogen peroxide. Sci. Rep..

[109] Skalickova S., Loffelmann M., Gargulak M., Kepinska M., Docekalova M., Uhlirova D., Stankova M., Fernandez C., Milnerowicz H., Ruttkay-Nedecky B. (2017). Zinc-Modified nanotransporter of doxorubicin for targeted prostate cancer delivery. Nanomaterials.

[110] Prochazkova S., Vårum K.M., Ostgaard K. (1999). Quantitative determination of chitosans by ninhydrin. Carbohydr. Polym..

[111] Leane M., Nankervis R., Smith A., Illum L. (2004). Use of the ninhydrin assay to measure the release of chitosan from oral solid dosage forms. Int. J. Pharm..

[112] Lucarelli G., Fanelli M., Larocca A.M.V., Germinario C.A., Rutigliano M., Vavallo A., Selvaggi F.P., Bettocchi C., Battaglia M., Ditonno P. (2012). Serum sarcosine increases the accuracy of prostate cancer detection in patients with total serum PSA less than 4.0 ng/mL. Prostate.

[113] Hurst R., Hooper L., Norat T., Lau R., Aune D., Greenwood D.C., Vieira R., Collings R., Harvey L.J., Sterne J.A. (2012). Selenium and prostate cancer: Systematic review and meta-analysis. Am. J. Clin. Nutr..

[114] Nishiya Y., Toda A., Imanaka T. (1998). Gene cluster for creatinine degradation in *Arthrobacter* sp. TE1826. Mol. Gen. Genet..

[115] Nishiya Y., Yamamoto M., Takemoto J.-i., Kano S., Nakano S. (2016). Monomeric sarcosine oxidase exhibiting high substrate affinity and thermostability. Int. J. Anal. Bio Sci..

[116] Gao C., Zhu H., Chen J., Qiu H. (2017). Facile synthesis of enzyme functional metal-organic framework for colorimetric detecting H_2_O_2_ and ascorbic acid. Chin. Chem. Lett..

[117] Mateo D., Morales P., Ávalos A., Haza A.I. (2014). Oxidative stress contributes to gold nanoparticle-induced cytotoxicity in human tumor cells. Toxicol. Mech. Methods.

[118] Smith M.R., Boenzli M.G., Hindagolla V., Ding J., Miller J.M., Hutchison J.E., Greenwood J.A., Abeliovich H., Bakalinsky A.T. (2013). Identification of gold nanoparticle-resistant mutants of Saccharomyces cerevisiae suggests a role for respiratory metabolism in mediating toxicity. Appl. Environ. Microbiol..

[119] Pokharkar V., Dhar S., Bhumkar D., Mali V., Bodhankar S., Prasad B. (2009). Acute and subacute toxicity studies of chitosan reduced gold nanoparticles: A novel carrier for therapeutic agents. J. Biomed. Nanotech..

[120] Thanou M., Verhoef J., Junginger H. (2001). Oral drug absorption enhancement by chitosan and its derivatives. Adv. Drug Dev. Rev..

[121] Richardson S.W., Kolbe H.J., Duncan R. (1999). Potential of low molecular mass chitosan as a DNA delivery system: Biocompatibility, body distribution and ability to complex and protect DNA. Int. J. Pharm..

[122] Santhosh S., Sini T.K., Anandan R., Mathew P.T. (2007). Hepatoprotective activity of chitosan against isoniazid and rifampicin-induced toxicity in experimental rats. Eur. J. Pharmacol..

[123] Kong M., Chen X.G., Xing K., Park H.J. (2010). Antimicrobial properties of chitosan and mode of action: A state of the art review. Int. J. Food Microbiol..

[124] Dutta P., Tripathi S., Mehrotra G., Dutta J. (2009). Perspectives for chitosan based antimicrobial films in food applications. Food Chem..

[125] Gupta D., Haile A. (2007). Multifunctional properties of cotton fabric treated with chitosan and carboxymethyl chitosan. Carbohydr. Polym..

[126] Helander I., Nurmiaho-Lassila E.-L., Ahvenainen R., Rhoades J., Roller S. (2001). Chitosan disrupts the barrier properties of the outer membrane of Gram-negative bacteria. Int. J. Food. Microbiol..

[127] Yang T.-C., Chou C.-C., Li C.-F. (2005). Antibacterial activity of *N*-alkylated disaccharide chitosan derivatives. Int. J. Food Microbiol..

[128] Neun B.W., Dobrovolskaia M.A. (2011). Method for analysis of nanoparticle hemolytic properties in vitro. Characterization of Nanoparticles Intended for Drug Delivery.

[129] Dobrovolskaia M.A., Clogston J.D., Neun B.W., Hall J.B., Patri A.K., McNeil S.E. (2008). Method for analysis of nanoparticle hemolytic properties in vitro. Nano Lett..

[130] Shen M., Cai H., Wang X., Cao X., Li K., Wang S.H., Guo R., Zheng L., Zhang G., Shi X. (2012). Facile one-pot preparation, surface functionalization, and toxicity assay of APTS-coated iron oxide nanoparticles. Nanotechnology.

[131] Jentzmik F., Stephan C., Lein M., Miller K., Kamlage B., Bethan B., Kristiansen G., Jung K. (2011). Sarcosine in prostate cancer tissue is not a differential metabolite for prostate cancer aggressiveness and biochemical progression. J. Urol..

[132] Cernei N., Zitka O., Ryvolova M., Adam V., Masarik M., Hubalek J., Kizek R. (2012). Spectrometric and electrochemical analysis of sarcosine as a potential prostate carcinoma marker. Int. J. Electrochem. Sci..

[133] Jiang Y., Cheng X., Wang C., Ma Y. (2010). Quantitative determination of sarcosine and related compounds in urinary samples by liquid chromatography with tandem mass spectrometry. Anal. Chem..

[134] Meyer T.E., Fox S.D., Issaq H.J., Xu X., Chu L.W., Veenstra T.D., Hsing A.W. (2011). A reproducible and high-throughput HPLC/MS method to separate sarcosine from α-and β-alanine and to quantify sarcosine in human serum and urine. Anal. Chem..

[135] Wu H., Liu T., Ma C., Xue R., Deng C., Zeng H., Shen X. (2011). GC/MS-based metabolomic approach to validate the role of urinary sarcosine and target biomarkers for human prostate cancer by microwave-assisted derivatization. Anal. Bional. Chem..

[136] Biavardi E., Tudisco C., Maffei F., Motta A., Massera C., Condorelli G.G., Dalcanale E. (2012). Exclusive recognition of sarcosine in water and urine by a cavitand-functionalized silicon surface. Proc. Nat. Acad. Sci. USA.

[137] Bianchi F., Dugheri S., Musci M., Bonacchi A., Salvadori E., Arcangeli G., Cupelli V., Lanciotti M., Masieri L., Serni S. (2011). Fully automated solid-phase microextraction–fast gas chromatography–mass spectrometry method using a new ionic liquid column for high-throughput analysis of sarcosine and *N*-ethylglycine in human urine and urinary sediments. Anal. Chim. Acta.

[138] Lan J., Xu W., Wan Q., Zhang X., Lin J., Chen J., Chen J. (2014). Colorimetric determination of sarcosine in urine samples of prostatic carcinoma by mimic enzyme palladium nanoparticles. Anal. Chim. Acta.

[139] Sabnis S., Block L.H. (2000). Chitosan as an enabling excipient for drug delivery systems: I. Molecular modifications. Int. J. Biol. Macromol..

[140] Owen J., Iggo B., Scandrett F., Stewart C. (1954). The determination of creatinine in plasma or serum, and in urine; a critical examination. Biochem. J..

